# A Leader-Guided Substrate Tolerant RiPP Brominase Allows Suzuki–Miyaura Cross-Coupling Reactions for Peptides and Proteins

**DOI:** 10.1021/acs.biochem.3c00222

**Published:** 2023-06-05

**Authors:** Nguyet A. Nguyen, Vinayak Agarwal

**Affiliations:** † School of Chemistry and Biochemistry, Georgia Institute of Technology, Atlanta, Georgia 30332, United States; ‡ School of Biological Sciences, Georgia Institute of Technology, Atlanta, Georgia 30332, United States

## Abstract

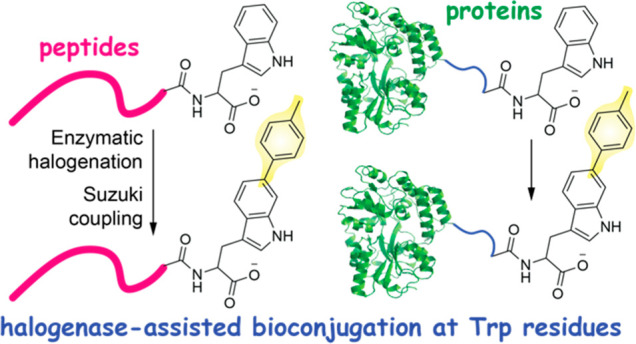

Bioorthogonal derivatization of peptides and proteins enables investigations into their biological function and allows for exploitation of their therapeutic potential, among other varied deliverables. Herein, we describe a marine halogenating enzyme-assisted bioconjugation strategy in which an N-terminal leader peptide guides bromination of a C-terminal Trp residue in genetically encoded peptides and proteins, setting up further Trp arylation by Suzuki–Miyaura reactions. The bromination and subsequent cross-coupling reactions are residue-specific and regiospecific for the indole-6 position, occur under mild aqueous conditions, and do not require any modification of other Trp residues in the substrate peptide and/or protein. Workflows described herein demonstrate the applicability of halogenating enzymes in bioorthogonal conjugation chemistry.

Suzuki–Miyaura cross-coupling is a universal palladium-assisted carbon–carbon bond-forming reaction typically involving aryl halides and organoboron substrates that enables the bioorthogonal derivatization of peptides and proteins.^1−3^ With an inventory of organoboron reaction partners already available,^4^ the key consideration is the preparation of the peptidic aryl halides. Reported strategies for introducing halogens into peptides and proteins include expanding the genetic code to incorporate amino acids with halogenated side chains,^5,6^ post-translational chemical modifications such as the alkylation of cysteine side chains to generate aryl halide thioethers,^7,8^ and chemical synthesis of the peptidic substrates with preinstalled halogen handles.^9,10^ For short synthetic peptides,^11^ enzymatic halogenation of indolic and phenolic rings sets up subsequent derivatization via Suzuki–Miyaura coupling.^12,13^ However, enzymatic halogenation of genetically encoded peptides and proteins has been out of reach as the repertoire of enzymes that halogenate peptidic substrates is limited.

Biocatalytic halogenation is rooted in natural product biosynthetic enzymology.^14^ The flavin-dependent halogenase MibH that is involved in the biosynthesis of the ribosomally synthesized and post-translationally modified lanthipeptide antibiotic NAI-107 is a regiospecific tryptophan side chain chlorinase.^15^ MibH is substrate selective; MibH chlorinated the Trp side chain indole only when all other post-translational modifications had been installed upon the NAI-107 precursor peptide, MibA, including the proteolytic removal of the modified core region from the MibA leader (Figure 1A).

**Figure 1:**
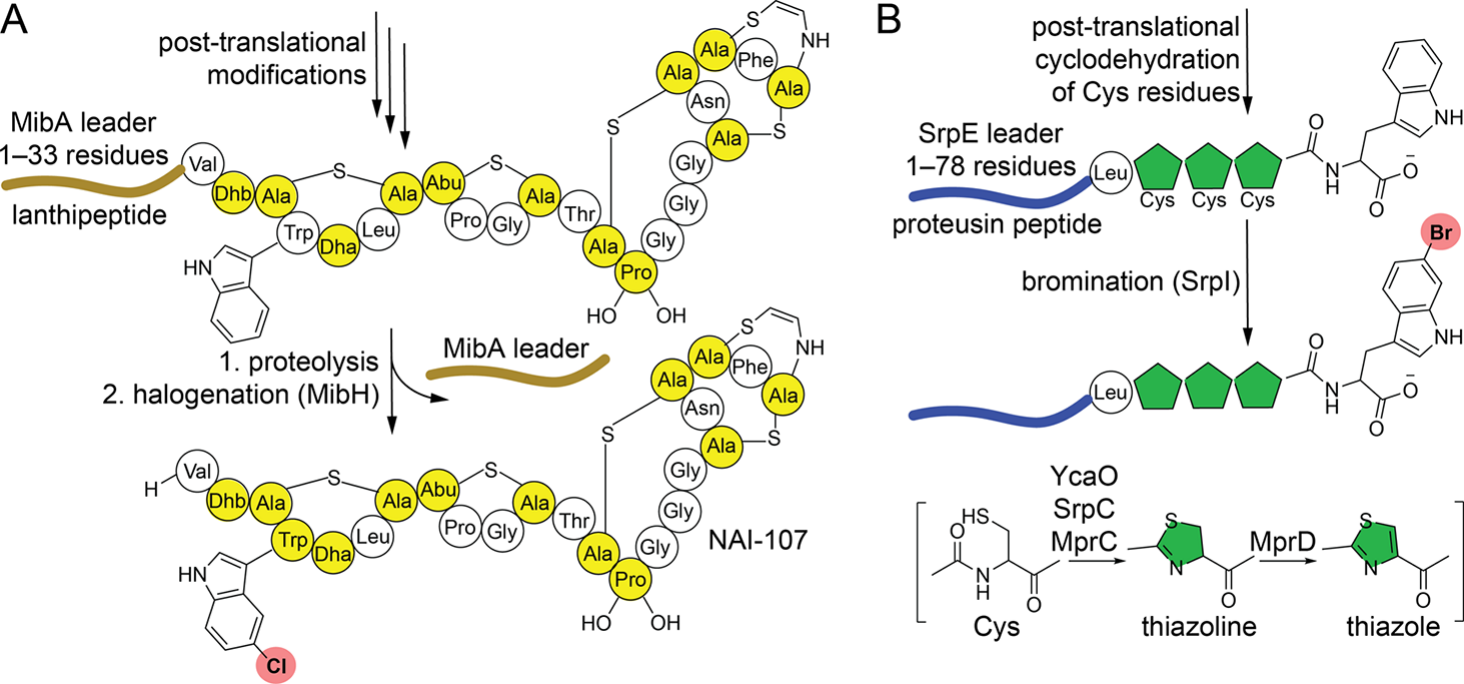
(A) Biosynthesis of lanthipeptide NAI-107. The 33-residue N-terminal leader sequence of the MibA substrate peptide guides post-translational modification of the C-terminal core. Proteolytic removal of the MibA leader is followed by halogenation by MibH. Post-translationally modified residues are colored yellow. Abbreviations: Dbh, dehydrobutyrine; Dha, dehydroalanine; Abu, aminobutyric acid. (B) The YcaO cyclodehydratase SprC catalyzes the cyclodehydration of three Cys residues to thiazoline in the SrpE substrate peptide. Bromination of the C-terminal Trp side chain is affected by SrpI.

We recently described a peptide brominase, SrpI, encoded in the microbiomes of marine sponges.^16^ The likely physiological substrate for the SrpI was the SrpE peptide in which the three Cys residues in the SrpE core, -LCCCW, were modified into thiazoline rings by the YcaO cyclodehydratase SrpC (Figure 1B). Preparation of the post-translationally modified SrpI substrate, and its derivatives, was hampered by the poor activity and strict substrate selectivity of the SrpC. Recombinant SrpC was not amenable to purification, and co-expression of *srpC* and *srpE* genes in *Escherichia coli* yielded a mixture of partially modified SrpE products. This observation in turn precluded the *in vitro* activity reconstitution of SrpI, evaluation of its the substrate scope, querying whether the activity of SrpI was dependent on the SrpE leader, and realizing the potential of SrpI as a general-purpose biocatalyst for peptide and protein halogenation.

To address the challenge of the preparation of the physiological substrate for SrpI, we turned to the substrate promiscuous YcaO cyclodehydratase/azoline oxidase pair MprC/MprD that we had described for installing azol(in)e heterocycles into 10 different MprE substrate peptides (Figure 1B and Figure S1).^17^ MprE and SrpE are proteusin peptides, characterized by long leader sequences that are similar to those of nitrile hydratases.^18^ While the MprC/MprD demonstrated robust activities *in vivo* and *in vitro*, they were selective for the MprE leader sequences; in contrast, SrpI was tolerant to other proteusin leaders.^16,17^ In light of these observations, we appended the SrpE -LCCCW core to the MprE_X_ leader (a consensus leader sequence built from the 10 different MprE leader sequences^17^), thus creating a chimeric MprE_X_–LCCCW substrate (Supplementary Note). Upon co-expression of the gene encoding this chimeric substrate with *mprC*/*mprD* in *E*. *coli*, we obtained the purified MprE_X_–LCCCW peptide in which all three Cys residues in the core were neatly converted into thiazol(in)e heterocycles (Figure 2A and Figure S2). Using the thusly prepared substrate, the brominating activity of purified flavin-dependent brominase SrpI was successfully reconstituted *in vitro* when paired with the flavin reductase RebF and the phosphite dehydrogenase PTDH (Figure 2B and Figure S3).^19,20^ To mitigate potential cross reactivity with hydrogen peroxide that is produced when flavin cofactor redox cycling is uncoupled from halide oxidation,^21^ catalase was included in all *in vitro* reactions.

**Figure 2:**
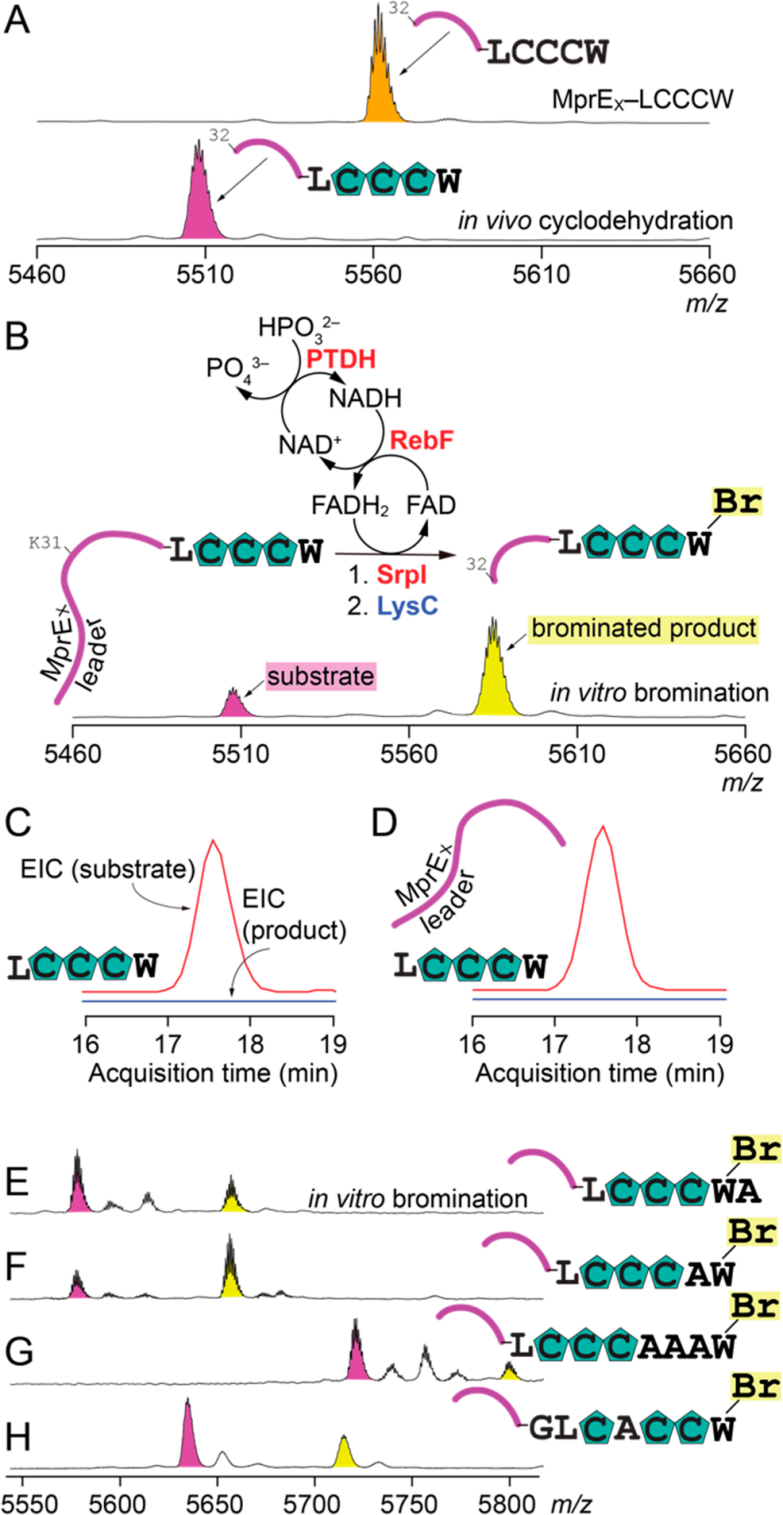
(A) MALDI-ToF MS spectra for the unmodified MprE_X_–LCCCW chimeric peptide (top) and the MprE_X_–LCCCW peptide in which the three Cys residues were converted to thiazol(in)e heterocycles (bottom). Digestion with the LysC protease removes the N-terminal 31 amino acids from the MprE_X_ leader. (B) Reaction scheme for *in vitro* bromination by SrpI. LC/MS extracted ion chromatograms (EICs) demonstrating that bromination does not occur when (C) the modified LCCCW core is provided to SrpI by itself or (D) *in trans* with the dissociated MprE_X_ leader peptide. The EIC for the modified LCCCW core is colored red, and that for the conceivable brominated product blue. The retention time for the brominated product lies within the acquisition time window illustrated here (Figure S6). (E–H) Bromination of modified cores appended to the MprE_X_ leader.

Establishing an *in vitro* assay allowed us to evaluate several aspects of the SrpI activity. First, we queried the leader peptide dependence for SrpI. When the modified LCCCW core was excised from the MprE_X_ core using the LahT150 peptidase^22^ and provided by itself as a substrate to SrpI without the MprE_X_ leader, no bromination was observed (Figure 2C). When the modified LCCCW core was provided *in trans* with the dissociated MprE_X_ leader, as before, no bromination of the core was observed (Figure 2D). SrpI did not brominate free tryptophan either (Figure S4). While bromination of indole was observed, bromination regiospecificity changed from SrpI being an indole-6 brominase for RiPP substrates to halogenating position 3 of free indole (Figure S5).^16^ Collectively, these data allow us to posit that the presence of the proteusin leader is an obligate requirement for the SrpI brominase. Both leaders, SrpE and MprE_X_, support SrpI activity. This inference is in contrast to the RiPP chlorinase MibH that catalyzes tryptophan chlorination only after the MibA core has been removed from the MibA leader.^15^

We next evaluated the selectivity of SrpI for different core sequences. Conservative modifications in which an alanine residue was added after and before the terminal Trp residue in the LCCCW core were tolerated by SrpI, yielding brominated products in each case (Figure 2E,F and Figures S7 and S8). A tripeptide extension before the terminal Trp residue was also tolerated (Figure 2G and Figure S9). However, a tripeptide extension after the Trp residue (MprE_X_–LCCCWAAA) was not processed by SrpI (Figure S10). Genes encoding all of the substrates mentioned above were co-expressed with *mprC*/*mprD* converting the Cys residues to thiazol(in)es. The consecutive thiazol(in)e sequence could be disrupted, and the MprE_X_–GLCACCW substrate was brominated (Figure 2H and Figure S11). Crucially, moving the Trp residue away from the C-terminus, substrates MprE_X_–GLCWCCC and MprE_X_–GLCAWCC, did not result in bromination by SrpI (Figures S12 and S13).

Data presented above identify two requirements for SrpI activity: the presence of a proteusin leader and the Trp residue being present at the C-terminus of the core. To test whether meeting these requirements allows for extension of the substrate scope of SrpI, we turned our attention to tumor-homing (TH) hexa- and heptapeptides. The TH peptides can deliver payloads specifically to tumor cells, making them attractive vehicles for the delivery of therapeutic payloads.^23^ We employed two TH peptides, here termed TH1 and TH2, LTVPLW and VLTVQPW, respectively, that possess terminal Trp residues.^24^ In contrast to the physiological pentapeptide substrate SrpE, the TH1 and TH2 peptides are hexa- and heptapeptides, respectively. Note that while SrpI can modify octapeptides, as well [substrate core LCCCAAAW (Figure 2G)], the observed substrate turnover was lower. In contrast to the physiological substrate, the TH peptides bear no azol(in)e heterocycles, though the Pro residues in the TH peptides could serve as surrogates for azol(in)e heterocycles in the substrate peptide core, as has been observed for other RiPP-modifying enzymes.^25,26^ The TH1 and TH2 sequences were appended to the SrpE proteusin leader. Bromination of these chimeric substrates was observed *in vitro* [for SrpE-TH2 (Figure 3A,B and Figure S14)] and *in vivo* upon co-expression of peptide-encoding genes with *srpI* [for SrpE-TH1 (Figure 3D,E and Figure S15)]. We also verified that the bromination of SrpE-TH1 proceeded *in vitro* in a time-dependent manner (Figure 3G). Despite the TH core sequences being divergent from the SrpE and bereft of azoline heterocycles, SrpI maintained regiospecificity for the terminal Trp bromination at the indole-6 position (Figure S16). SrpI also maintained its rigid specificity for bromination, and chlorination of either substrate was not observed *in vivo*, or *in vitro* (Figures S17 and S18). Though the Gln residue in TH2 was well tolerated, the current inventory of SrpI substrates generally consists of nonpolar peptides. An expanded investigation of the substrate scope of SrpI will involve investigating whether charged residues can also be accommodated in the substrate core.

**Figure 3:**
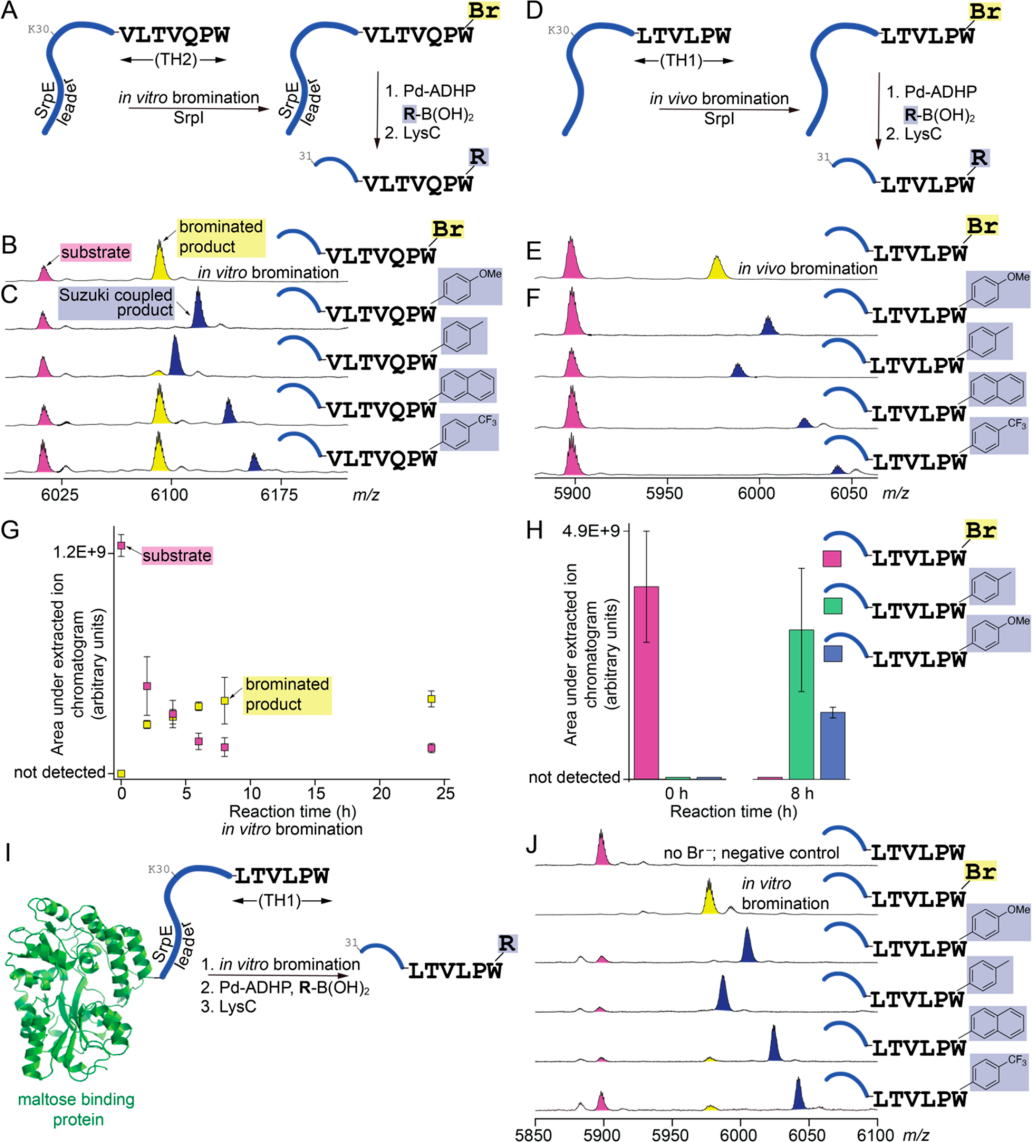
(A) Reaction scheme for *in vitro* bromination and Suzuki–Miyaura cross-coupling for SprE-TH2. Abbreviation: ADHP, 2-amino-4,6-dihydroxy-pyrimidine. (B and C) MALDI-ToF MS spectra demonstrating the unmodified peptide (pink peaks), brominated peptide (yellow peaks), and Suzuki–Miyaura coupling products (blue peaks) for the SprE-TH2 substrate. (D) Reaction scheme for *in vivo* bromination and Suzuki–Miyaura cross-coupling for SprE-TH1. (E and F) MALDI-ToF MS spectra demonstrating the unmodified peptide, brominated peptide, and coupling product for the SprE-TH1 substrate. (G) Time-dependent *in vitro* bromination of SrpE-TH1 by SrpI. Assays after proteolytic digestion were analyzed by liquid chromatography/mass spectrometry (LC/MS), and areas under the extracted ion chromatograms corresponding to the substrate peptide and the brominated product were determined. Means and standard deviations for three independent reactions are plotted. Note that the substrate and product peptides demonstrate disparate abundances using LC/MS. (H) Abundance of the brominated SrpE-TH1 peptide and after coupling to toluene and *p*-methoxyphenyl boronic acids monitored by LC/MS. (I) Reaction scheme for *in vitro* bromination and Suzuki–Miyaura cross-coupling for the SprE-TH1 peptide appended to the C-terminus of the maltose binding protein (illustrated as a green ribbon, Protein Data Bank entry 1FQD(27)). (J) MALDI-ToF MS spectra demonstrating a negative control reaction in which no bromide was added, and hence no brominated product was observed, followed by detection of the brominated and conjugated products.

In line with the extensive application of aryl halogenation as a reactive handle for late-stage chemical diversification,^10,12,13,28,29^ we explored the Suzuki–Miyaura coupling of a panel of boronic acids to brominated peptides furnished by SrpI. For both *in vitro*-brominated SrpE-TH2 and *in vivo*-brominated SrpE-TH1, peptides that are >100 amino acids in length, coupling to boronic acids was observed (Figure 3C,F and Figures S19–S26). Obligate bromination by SrpI, without contaminating chlorination, allowed for mild reaction conditions in aqueous buffer. Qualitatively, in this proof-of-concept demonstration, benzylic boronic acids with electron-donating substituents delivered a higher yield of cross-coupling products. This observation was corroborated by the stoichiometric yield for coupling toluene and *p*-methoxyphenyl boronic acids to the *in vitro*-brominated SrpE-TH1 peptide (Figure 3H). SrpI also enabled the bromination and Suzuki–Miyaura coupling on large globular proteins. The SrpE-TH1 sequence was appended at the C-terminus of the 400-residue maltose binding protein (Figure 3I). The chimeric protein was a competent substrate for *in vitro* bromination by SrpI, followed by Suzuki–Miyaura coupling under conditions that did not require protein denaturation or the use of organic co-solvents (Figure 3J and Figures S27–S31). It is noteworthy that the SrpI-mediated strategy for peptide and protein labeling did not require the mutation of other Trp residues; SrpI itself maintains specificity for labeling only the C-terminal Trp. The MprE_X_ leader possesses other Trp residues, as does the maltose binding protein, and they were not brominated and thus not conjugated.

While monitoring the halogenation assays mentioned above, we routinely observed the appearance of two brominated peptidic products, even in reactions in which the peptide/protein substrates were omitted. Using high-resolution mass spectrometry, we traced bromination to be occurring at two SrpI Tyr residues (SrpI Y102 and Y110); the brominated products mentioned above were generated by LysC digestion of SrpI in the reaction mixture (Figure 4A and Figure S32). Homology models indicate that these Tyr residues are proximal to the catalytic Lys residue (SrpI Lys84) that is implicated in forming a haloamine intermediate after halide oxidation at the flavin isoalloxazine ring or, as a proton donor, to facilitate resolution of the hypohalous acid intermediate (Figure S33).^21^ Mutating either or both these Tyr residues did not compromise SrpI activity (Figure 4B). Our fortuitous discovery of SrpI self-halogenation was enabled by monitoring the progress of SrpI reactions using MALDI-ToF MS; it is conceivable that the self-halogenation could occur for other halogenating enzymes that bear electron rich amino acid side chains near the catalytic Lys residue.

**Figure 4:**
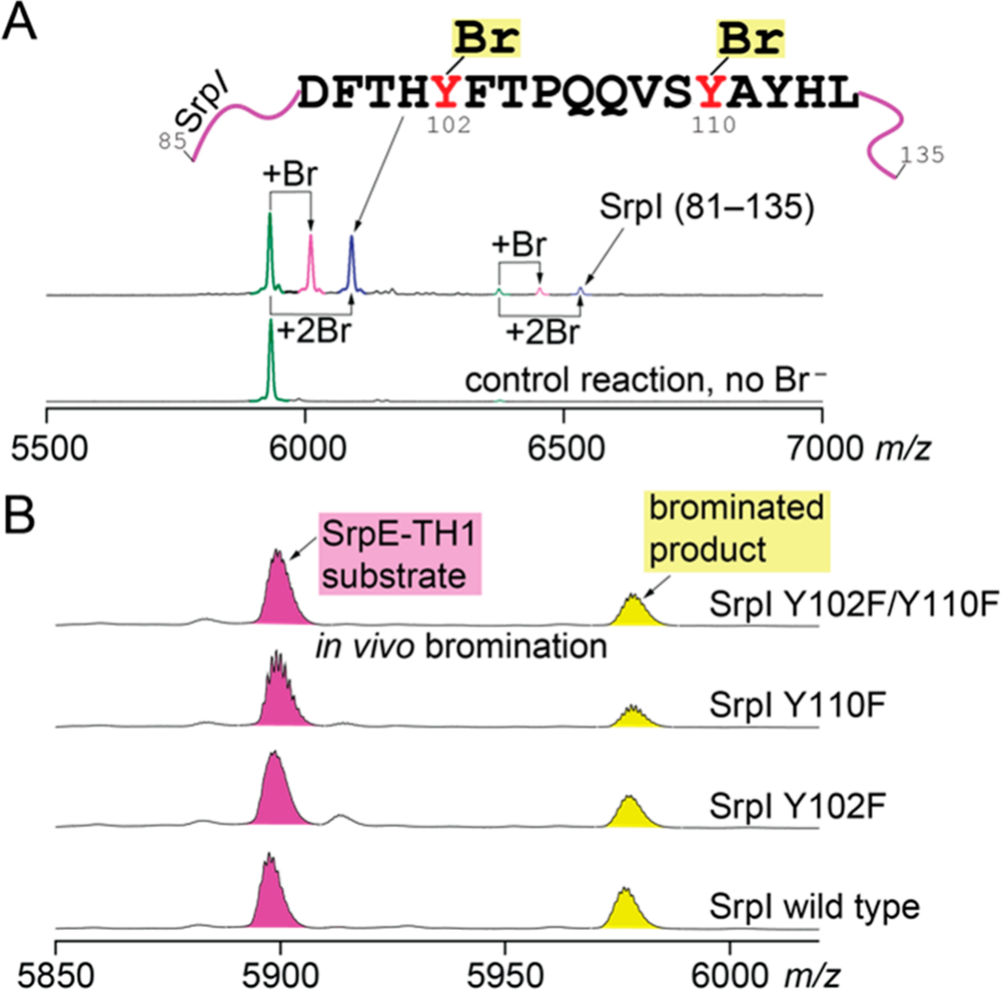
(A) MALDI-ToF MS spectra demonstrating the detection of mono- and dibrominated derivatives of SrpI 85–135 and SrpI 81–135 peptides generated by LysC digestion of SrpI. Self-bromination occurs at Tyr102 and Tyr110 residues. Bromide was omitted from the control reaction. (B) Bromination activity of wild type and mutant SrpI enzymes evaluated by *in vivo* bromination of the SrpE-TH1 chimeric substrate.

Flavin-dependent halogenases have previously been used for the halogenation of short synthetic peptides containing tryptophan residues.^11^ However, to the best of our knowledge, SrpI represents the first of its class enzyme for a leader peptide-guided bromination of genetically encoded peptides. The ability to deliver a bromide adduct selectively upon a single Trp residue in a ribosomally translated peptide and protein presents the opportunity to further develop SrpI as a biotechnology tool to facilitate bioorthogonal Suzuki–Miyaura cross-coupling reactions, and other conjugation reactions requiring halogenated peptide/protein precursors. These efforts will require an expanded investigation of the substrate scope for SrpI, as well as confirmation that brominating a fusion peptide at the C-terminus of a protein substrate does not alter the function or activity of the protein itself. As such, because the activity of SrpI is restricted to C-terminal residues and does not extend to internal Trp residues, SrpI likely serves to provide a route for peptide/protein labeling and bioconjugation, rather than modulation of the structure and activity of the biomolecular substrate.

Compared to amino acids such as Cys and Lys, chemical strategies for Trp arylation are sparse and almost exclusively restricted to position 2 of indole.^8^ The regiospecificity of SrpI, bromination at indole-6, opens other sites on the indole side chain for modification. Several bottlenecks need to be overcome to improve the applicability of SrpI as a biocatalyst, among which is the limited activity of SrpI observed *in vivo*, contracting the leader peptide required for SrpI activity, and ameliorating the deleterious consumption of oxidized bromine for self-halogenation of Tyr residues. Though in line with previous reports for cross-coupling reactions with peptidic substrates,^30−33^ the organometallic catalyst loading in our reactions is currently high. A screening of organoboron reaction partners (Figure S34), core peptides of different lengths bearing the terminal Trp residue, and reaction conditions is currently underway.

## Supporting Information Available

Experimental details for recombinant protein production, synthetic procedures, analytical procedures for compound characterization, Figures S1–S34, a supplementary note describing chimeric peptide sequences used in this study, and supplementary references (PDF)

## Accession Codes

The SrpI and SrpE sequences are available from GenBank using the BioProject number PRJNA694437 and have been added to the supplementary note.
