# Supplementary material for: A Leader-Guided Substrate Tolerant RiPP Brominase Allows Suzuki–Miyaura Cross-Coupling Reactions for Peptides and Proteins
Source: Biochemistry. 2023 Jun 5;62(12):1838–43. doi: 10.1021/acs.biochem.3c00222 (PMC10286304; doi:10.1021/acs.biochem.3c00222)
Supplement: Supplementary file 1 — bi3c00222_si_001.pdf [file bi3c00222_si_001.pdf]

SUPPLEMENTARY INFORMATION FOR:

A leader-guided substrate tolerant RiPP brominase allows Suzuki–Miyaura cross-coupling reactions for peptides and proteins

Nguyet A. Nguyen,[1] and Vinayak Agarwal[1,2,*]

[1]School of Chemistry and Biochemistry, Georgia Institute of Technology, Atlanta, GA 30332, USA

[2]School of Biological Sciences, Georgia Institute of Technology, Atlanta, GA 30332, USA

*Correspondence: vagarwal@gatech.edu; Ph: (+1)404-385-378

SUPPLEMENTARY MATERIALS AND METHODS

Preparation of constructs for expression of SrpI, PtdH, RebF, and chimera peptides

Genes optimized for expression in *Escherichia coli* for SrpI, PtdH, RebF, and the chimeric substrate peptides were used as templates for the amplification and subcloning of PCR amplicons in plasmid vectors. The chimeric peptides were designed by incorporating the leader peptide SrpE or the consensus leader peptide sequence $MprE_X$ as described in the Supplementary Note (below).

General polymerase chain reaction protocol

PCR reactions (25 µL) contained 20 ng template DNA, 0.4 µM each of reverse and forward primers, 0.2 mM dNTPs, Phusion reaction buffer, and 0.25 U Phusion-high fidelity DNA polymerase (Thermo). PCR amplicons were subcloned into plasmid vectors using Gibson Assembly HiFi master mix (NEB). For co-expression with chimeric substrate peptide genes, the modification enzyme encoding genes (*mprC*, *mprD*, and *srpI*) were subcloned without any affinity chromatography tags. All constructs were verified by Sanger sequencing.

Protein expression and purification

General procedure for heterologous protein expression: Plasmid DNA (20 ng) was transformed in *E. coli* BL21(DE3). Colonies were grown under appropriate antibiotic selection on LB agar media for 16 h. A single colony was picked and inoculated in 10 mL of terrific broth (TB) supplemented with appropriate antibiotics for 16 h at 37 °C. This inoculum was used to initiate 1 L TB cultures supplemented with corresponding antibiotics. Cultures were incubated with shaking at 30 °C until the OD600 reached 0.6. Cultures were cooled at 18 °C for 1 h before induction of protein expression by adding 0.3 mM isopropyl-β-d-thiogalactopyranoside (IPTG). Cultures were incubated at 18 °C, 180 rpm for 24 h.

General purification protocol for N-His_6-substrate peptides: Cultures were harvested by centrifugation (5,000 rpm, 25 min, 4 °C) and resuspended in 50 mL lysis buffer A (20 mM Na-phosphate (pH 7.5), 50 mM NaCl, 4M Gdn-HCl). Cells were lysed by sonication, and the lysate was clarified by centrifugation at 18,000 rpm for 45 min at 4 °C. The supernatant was loaded onto a 5 mL His-Trap Ni-NTA column equilibrated with the lysis buffer. The column was washed with 10 mL wash buffer A1 (20 mM Na-phosphate (pH 7.5), 50 mM NaCl, 30 mM imidazole, 4M Gdn-HCl), 10 mL of 1:1 wash buffer A1:wash buffer A2 (20 mM Na-phosphate (pH 7.5), 50 mM NaCl, 30 mM imidazole), and 10 mL wash buffer A2. Bound proteins were then eluted using elution buffer A (20 mM Na-phosphate (pH 7.5), 50

mM NaCl, 1M imidazole) in three fractions of 5 mL volume each. The second fraction was found to contain the highest concentration of the peptide, and thus, collected. This fraction was desalted using Sephadex G-25 PD10 column in storage buffer B (20 mM Na-phosphate (pH 7.5), 100 mM NaCl). The peptide concentration was measured by Bradford assay. Aliquots were frozen and stored at –80 °C for future use.

Purification of PtdH and RebF was performed according to published protocols.[1-2]

Purification of N-His_6-SrpI: Chaperones (from chaperone plasmid pGro7, Takara) were co-expressed to assist the folding of SrpI. After addition of IPTG and 48 h of incubation at 18 °C, cultures were harvested by centrifugation as above, and cell pellets were resuspended in lysis buffer B (20 mM Na-phosphate (pH 7.5), 100 mM NaCl). Cells were lysed by sonication. The lysate was clarified by centrifugation at 18,000 rpm for 60 min. The supernatant was loaded onto a 5 mL His-Trap Ni-NTA column. The column was washed extensively with wash buffer B (20 mM Na-phosphate (pH 7.5), 100 mM NaCl, 30 mM imidazole), and protein was eluted using a linear gradient from 0% to 100% elution buffer B (20 mM Na-phosphate (pH 7.5), 100 mM NaCl, 250 mM imidazole). The purity of eluent fractions was checked by SDS-PAGE, and fractions containing protein of interest were pooled and concentrated by Amicon® Ultra Centrifugal Filter Unit. The protein was further purified by size exclusion chromatography on a Superdex 75 16/200 column with 20 mM Na-phosphate (pH 7.5), 100 mM NaCl buffer. The purity of eluent fractions was checked by SDS-PAGE and pure fractions pooled. The concentrations were measured by Bradford assay. Aliquots were frozen and stored at –80 °C for future use.

Enzymatic and chemo-enzymatic assays

Halogenation assays: Bromination reactions were performed in 200 µL volume containing 50 mM HEPES-Na (pH 7.5), 20 mM KBr, 25 µM FAD, 0.625 mM NAD^+, 6.25 mM Na_2HPO_3, 5 µM flavin reductase (RebF), 5 µM phosphite dehydrogenase (PTDH), 100 µM substrate peptide, 20 µM SrpI, 0.05 µg/µL catalase. After 24 h incubation at 30 °C, reactions were quenched by protease addition (LysC, LahT150, or carboxypeptidase A) according to published protocols.[3] The reactions treated with LysC were then desalted and analyzed by MALDI-ToF, the reactions treated with LahT150 and carboxypeptidase A were analyzed by HPLC-MS/MS.

Indole was used as a substrate at a final concentration of 2 mM in the above-mentioned assay. After 24 h of incubation, an equal volume of EtOAc was used to extract the assay. The mixture was then centrifuged at 16,000 rpm for 20 min to remove debris. The EtOAc extract was analyzed by GC-MS (1260G with 7890a MS; Agilent Technologies) in electron ionization (70 eV) mode using a DF-5ms ultra inert GC column (30 m length, 0.25 mm width and 0.5 μM film thickness). The column temperature conditions were as follows: 40 °C for 3 min, increased to 200 °C at 10 °C/min, and held for 1 min with a total run time of 20 min. The injection port, interface, and ion source were kept at 250 °C, 300 °C, and 230 °C, respectively. Helium was used as carrier gas at a 0.9 mL/min flow rate.

Time-course experiments to monitor the bromination of SrpE-TH1: The experimental procedure was conducted with a total volume of 600 μL, comprising a mixture of 50 mM HEPES-Na (pH 7.5), 20 mM KBr, 25 μM FAD, 0.625 mM NAD^+, 6.25 mM Na_2HPO_3, 5 μM flavin reductase (RebF), 5 μM phosphite dehydrogenase (PTDH), 20 μM substrate peptide SrpE-TH1, 10 μM SrpI, and 0.05 μg/μL catalase. To ensure optimal O_2 transfer, 100 μL assay was transferred and incubated in individual tubes. At 0, 2, 4, 6, 8, and 24 h, the reaction was quenched by adding 1 μL of 6N HCl, followed by brief centrifugation to remove precipitated protein. The reaction mixture was neutralized with 1 μL of 6N NaOH, and then 1 μL of 2 mg/mL GluC was added and incubated at 30 °C for an additional 2 h. Any precipitation was removed by centrifugation after adding 100 μL of MeOH. Finally, 100 μL of the quenched reaction mixture was analyzed by HPLC-MS, and the area under the curve for the starting material and product was calculated using the extracted chromatogram for $[M+2H]^{2+}$ ion at *m/z* 564.33 and 604.28, respectively.

d-labeling of indole: 2.4 mg indole was dissolved in 500 μL D_2O and stirred at 150 ^{0}C overnight. The product was analyzed by NMR using a 400 MHz Bruker NMR showing that conversion of indole to indole-3-*d* progressed with 85% yield. ^{1}H-NMR (400 MHz, D_2O): δ 7.61 (dt, 1H), δ 7.45 (dt, 1H), δ 7.31 (s, 1H), δ 7.15 (ddd, 1H), δ 7.06 (ddd, 1H). The sample was used for enzymatic halogenation without further purification.

Suzuki-Miyaura cross-coupling reactions

Several water soluble ligands have been developed to facilitate Suzuki-Miyaura cross coupling reactions, including phosphine-based ligands[4-5] and nitrogen-based ligands[6-7] (Fig. S34). Nitrogen-based Pd-ligands containing the guanidine moiety have been shown to be less sensitive to air than the

phosphine-based counterparts. Intensive studies in developing mild protocol for cross-coupling reaction utilizing guanidine moiety-containing ligands for proteins were conducted by Davis,[7-9] Lin,[10] and Zhang.[11] In our proof-of-concept demonstration, we adapted procedures for Suzuki-Miyaura cross-coupling reactions from Davis.[9]

Preparation of Pd-ADHP: To 5 mL 0.1 M NaOH, 13 mg 2-amino-4,6-dihydroxy pyrimidine (0.1 mmol) was added, followed by 11 mg palladium acetate (0.05 mmol). The solution was stirred at 65 ºC for 30 min to yield 0.01 M catalyst solution.

Brominated peptide SrpE-TH1 was obtained by co-expression of the substrate peptide (SrpE-TH1) encoding gene and *srpI*. The brominated peptide was purified and stored in 20 mM Na-phosphate (pH 7.5), 100 mM NaCl buffer.

The halogenation of the chimeric substrates SrpE-TH2 and MBP-SrpE-TH1 was performed *in vitro* on a 10 mL-scale reaction as described above. The reaction solution was desalted by Sephadex G-25 PD10 column using 20 mM Na-phosphate (pH 7.5), 100 mM NaCl buffer to remove all cofactors that have been shown to have a deleterious effect on Pd-catalyzed cross-coupling reaction.[12]

The cross-coupling reactions were performed in 200 µL volume containing 250 µM halogenated substrate peptide or proteins, 2.5 mM Pd-ADHP, 75 mM Na_2HPO_4, and 2.5 mM boronic acid. The reaction was incubated at 45 °C for 8 h. 1 µL 0.2 mg/mL LysC or GluC proteases were added and then incubated at 30 °C for further 90 min. 100 µL of 5 µL/mL 3-mercaptopropionic acid was added to chelate the excess Pd. The reactions treated with LysC were analyzed by MALDI-ToF, and the reactions treated with GluC were analyzed by LC/MS, as described below.

Mass spectrometry

For matrix-assisted laser desorption-ionization-time of flight mass spectrometry (MALDI-ToF MS), reactions samples were desalted using C_{18} ZipTips (Sigma) and spotted on a MALDI target using 2 µL saturated sinapinic acid (Sigma) in 7:3:0.1 MeCN:H_2O:TFA solvent for analysis by a rapifleX MALDI-ToF mass spectrometer (Bruker Daltonics) in reflectron positive ionization mode. The data were analyzed using flexAnalysis software.

The Suzuki-Miyaura coupling reaction products, the bromotryptophan standards, and the excision product generated by carboxypeptidase A were analyzed using Vanquish Flex UHPLC (Thermo) coupled to a Q Exactive HF hybrid quadrupole-orbitrap mass spectrometer (Thermo). Mass spectrometry data were collected in the positive ionization mode in the mass range *m/z* 100–2000 Da. Samples were analyzed using Acquity UPLC BEH C_{18} 1.7 µm column (100×2.1 mm) at a flow rate of 0.4 mL/min, and the chromatographic separation was achieved using solvent A: H_2O + 0.1% (v/v) formic acid, and solvent

B: MeCN + 0.1% (v/v) formic acid. The chromatography profile was as follows: 5% solvent B from 0–0.5 min, linear gradient to 100% solvent B from 0.5–7 min, 100% solvent B from 7–10 min, linear gradient to 5% solvent B from 10–11 min, 5% solvent B from 11–12 min.

The halogenated peptides after the digestion with LahT150 were analyzed by Bruker amaZon SL ion trap mass spectrometer coupled to an Agilent 1260 HPLC. Mass spectrometry data were collected in the positive ionization mode in the mass range *m/z* 100–1000 Da. Chromatography was performed using Luna 5 µm C_{18} reversed-phase HPLC column (100×4.6 mm) at a flow rate of 0.5 mL/min; solvent A: H_2O + 0.1% v/v formic acid, solvent B: MeCN + 0.1% v/v formic acid. The chromatography elution profile was as follows: 5% solvent B from 0–5 min, linear gradient to 100% solvent B from 5–35 min, 100% solvent B from 35–40 min, linear gradient to 5% solvent B from 40–41 min, and 5% solvent B from 41–42 min.

SUPPLEMENTARY NOTE

The sequence of the $MprE_X$ leader peptide:

MNEEQMQQYSQIVAKCWADAEFKAKLIADPKATLAAEGIAVPDGIELRVLENTATTVNLVLPPPP§AEGELSDEDL‡GAVTGG†

The sequence of the SrpE leader peptide:

MRSGDDMLQHLVEKSALDADFRQQLLADPKSTISQELGISIPESMTIRVHESDMETVHLALPPDP§NLTEEQL‡EAISAG†

The double glycine motif† which marks the leader/core boundary, the proline-rich motif,§ and the YcaO binding motif‡ are underlined.

The sequence of the SrpI halogenase:

MIQPGSESLRKIAVIGRGTAGSLAAASVTRLHPDADHELHHIYDSRIPVIGVGEGSWPSLVQEVQQLTGL
PHETVQQRLKGTRKYGVAFEGWGRRGRDFTHYFTPQQVSYAYHLSADLLADMLHESSRARHIDAKVLDIA
RVDGGARVEFEGRAPERYDLVFDARGFPRELDTDEHIDISFIPTNTAVIRRCPAIVEEAAGPVLQHTYTR
AVARPHGWIFVIPLAVHTSYGYIFNRDVTGLDEVESDFDAFLETDGVPEFEQRAVLRFPNFVHRRIYDGA
VARIGNAAAFMEPLEATAIVSAQIQIGMVLKTRLGRSVEHLDRDAPAVNRFLVKNVLRYGLFVGWHYSCG
SRYDSPFWRFARDRTWPRYRSAADPAAVDCNALGEFDEMIRLLHQPVIDQGDWHRMCAVPLTSYAQMSQG
LGC

Supplementary Table

Table S1. Calculated and observed masses of substrate and product peptides in this study

Compound	Calculated mass	Observed mass
LCCCW	569.15	569.19
Br-LCCCW	647.06	647.06
3-Br-indole	194.97	194.86
Indole-3-*d*	118.06	117.96
LCCCWA	640.18	640.18
Br-LCCCWA	718.09	718.09
LCCCAW	640.18	640.18
Br-LCCCAW	718.09	718.09
LCCCAAAW	782.26	782.26
Br-LCCCAAAW	862.17	862.24
LCCCWAAA	5717.81	5717.52
GLCACCW	697.20	697.20
Br-GLCACCW	775.11	775.12
GLCWCCC	5720.78	5718.28
GLCAWCC	5688.81	5687.22
AISAGLTVLPW (TH1)	1127.65	1127.65
AISAGVLTVQPW (TH2)	1241.69	1241.70
Br-TH1	1205.55	1205.55
Br-TH2	1319.60	1319.59
4-Methoxyphenyl-TH1	1233.69	1233.69
4-Methylbenzene-TH1	1217.69	1217.69
2-Naphthalene-TH1	1253.69	1253.69
4-Trifluoromethylphenyl-TH1	1271.66	1271.66
4-Methoxyphenyl-TH2	1347.73	1347.73
4-Methylbenzene-TH2	1331.74	1331.74
2-Naphthalene-TH2	1367.74	1367.74
4-Trifluoromethylphenyl-TH2	1385.70	1385.71

Supplementary Figures

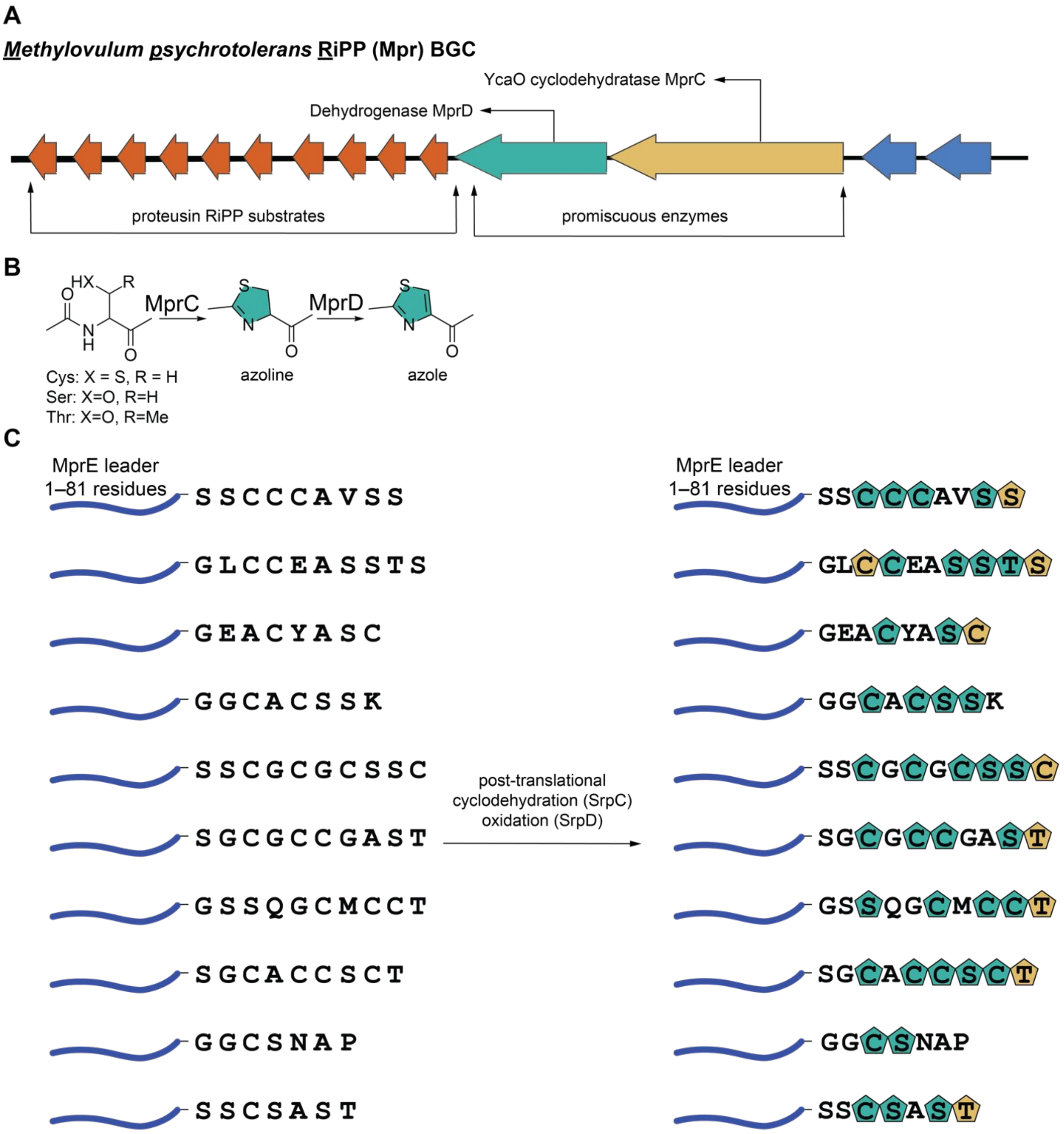

Fig. S1. (**A**) The *mpr* biosynthetic gene cluster showing the genes *mprC* and *mprD* gene clustered with genes encoding ten different MprE substrate peptides. (**B**) MprC catalyses the cyclodehydration of Cys, Ser, and Thr residues to form azoline rings. MprD catalyses the oxidation of azolines to azoles. (**C**) Promicuity activity of MprC/MprD allows for the installation of azol(in)e heterocycles in ten different MprE substrate peptides.

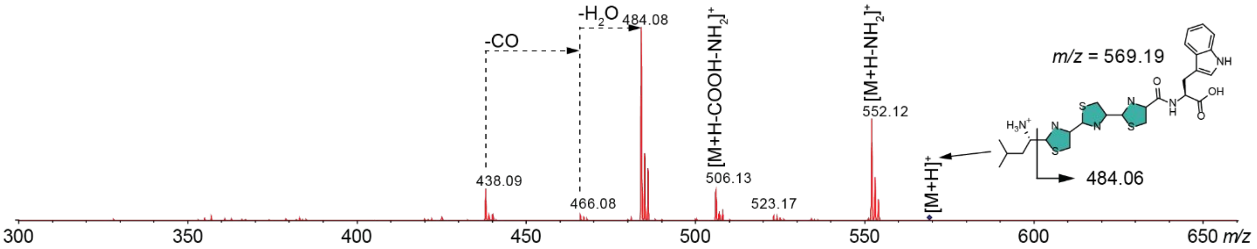

Fig. S2. Structural annotation of the MS^2 fragmentation spectra for the modified SrpE core (LCCCW) obtained by co-expression of the gene encoding the $MprE_X$-LCCCW chimeric substrate with *mprC and mprD*, followed by the treatment of the purified product with the protease LahT150 to remove the leader peptide and furnish the modified core as the product.

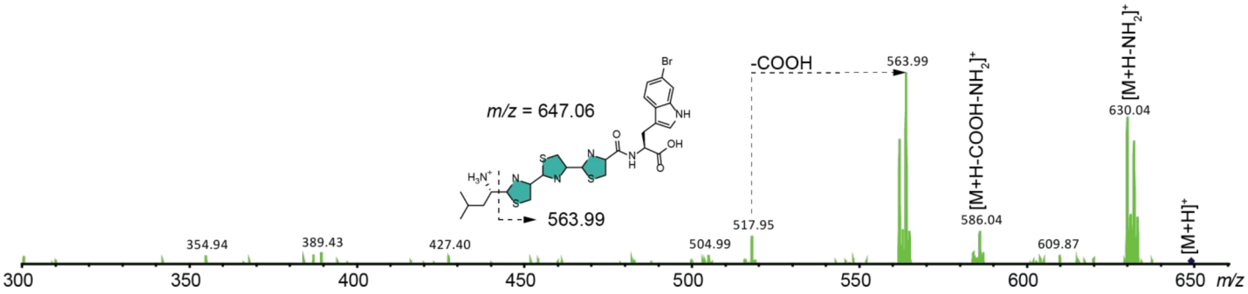

Fig. S3. Structural annotation of the MS^2 fragmentation spectra for the brominated SrpE core obtained by *in vitro* halogenation of modified $MprE_X$-LCCCW substrate by SrpI followed by the treatment of the product with LahT150.

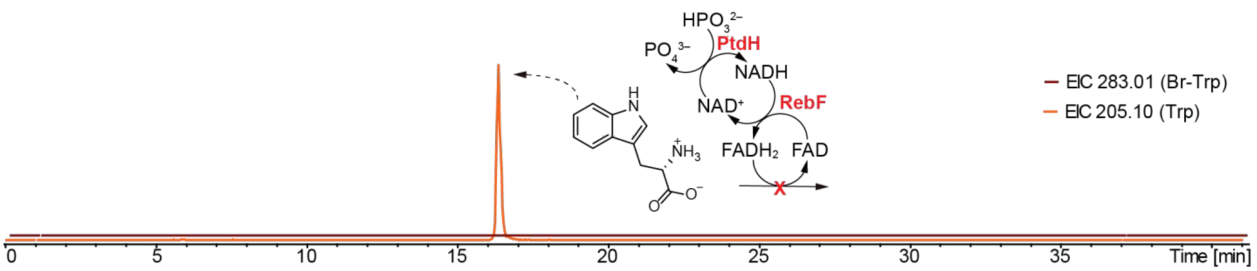

Fig. S4. LC-MS extracted ion chromatograms (EICs) for tryptophan and bromotryptophan showing that the bromination of free tryptophan is not catalyzed by SrpI.

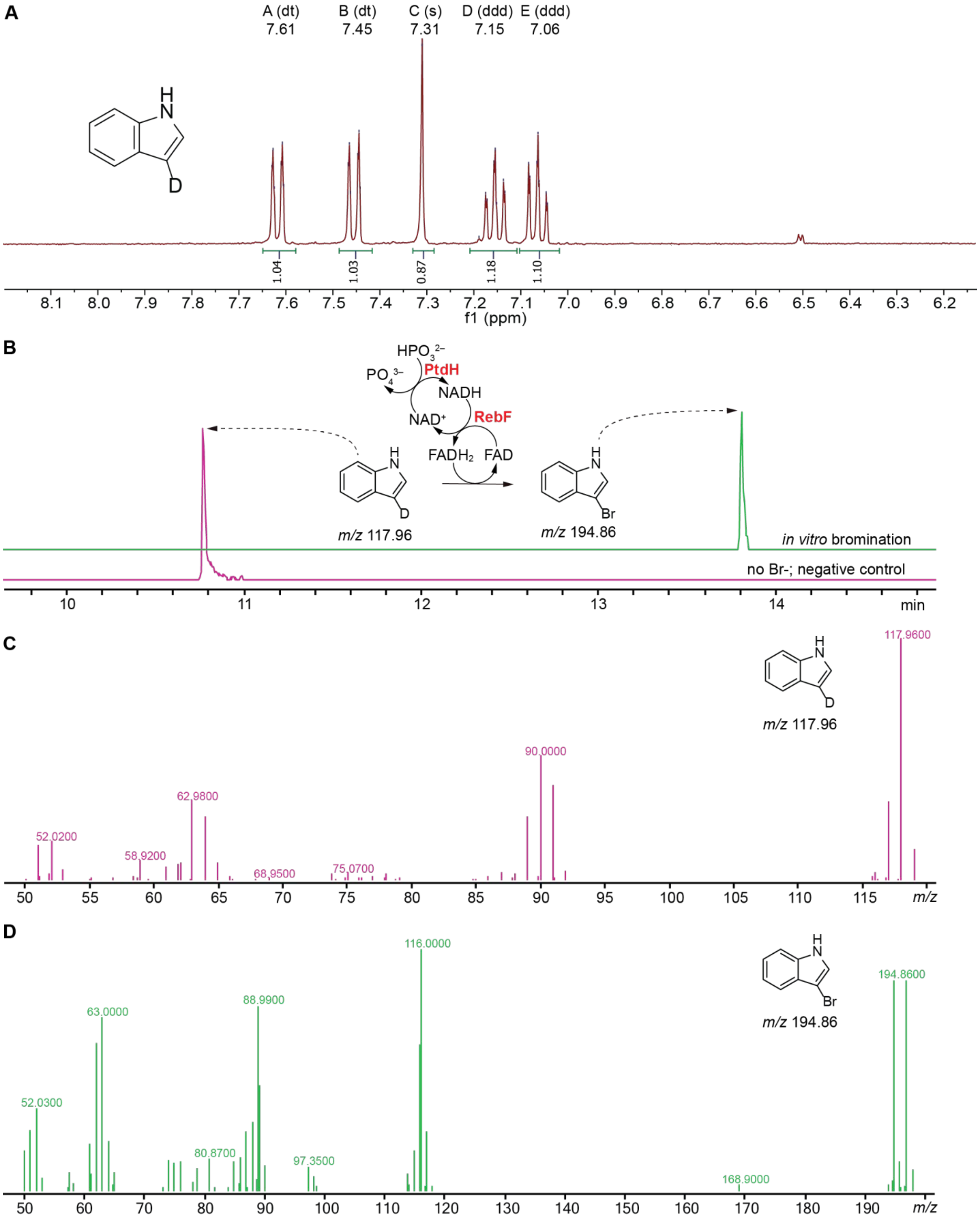

Fig. S5. (**A**) ^{1}H-NMR (400 MHz, D_2O) spectrum for indole-3-*d*. (**B**) GC-MS analysis for SrpI assays for indole-3-*d* showing the brominated product as 3-Br-indole. (**C**) MS spectra of indole-3-*d*. (**D**) MS spectra of 3-Br indole.

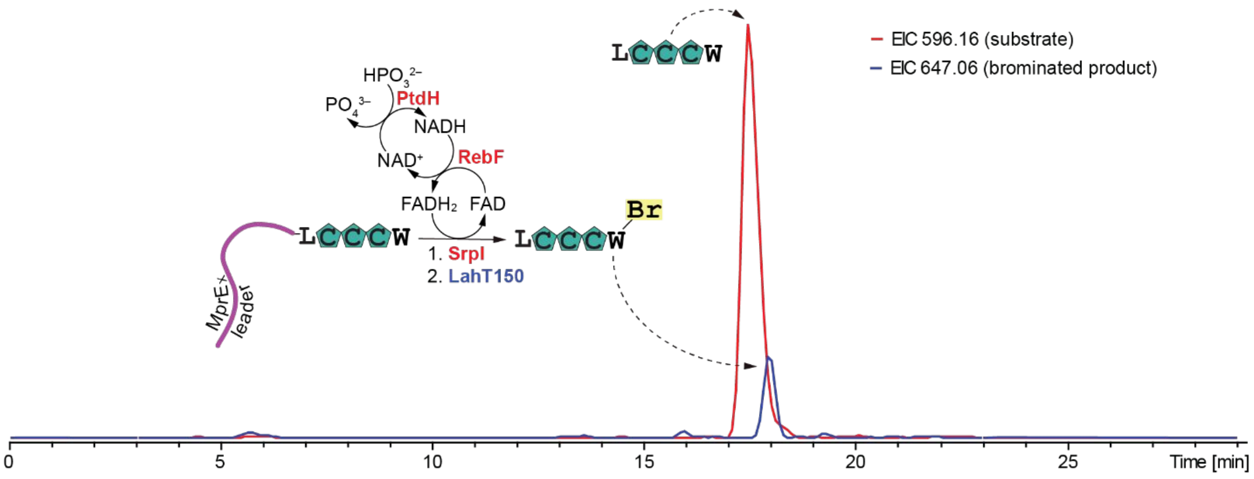

Fig. S6. Reaction scheme for *in vitro* bromination of the modified MprE$_X$-LCCCW chimeric substrate by SrpI followed by the treatment of the product with LahT150. LC-MS EICs demonstrate that the substrate and brominated products, after scission from the leader, are eluted within the time window from 16–19 min as is illustrated in Fig. 2C.

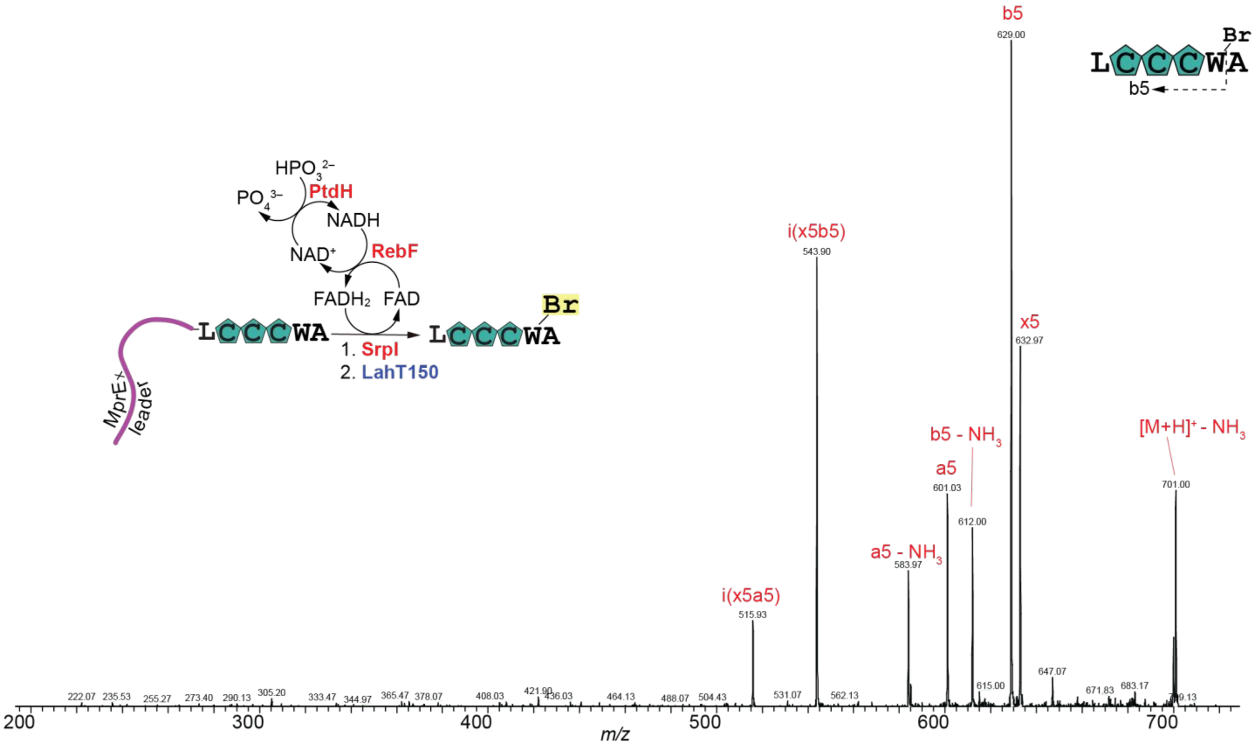

Fig. S7. Structural annotation of the MS^2 fragmentation spectra for the brominated L<u>CCC</u>WA core.

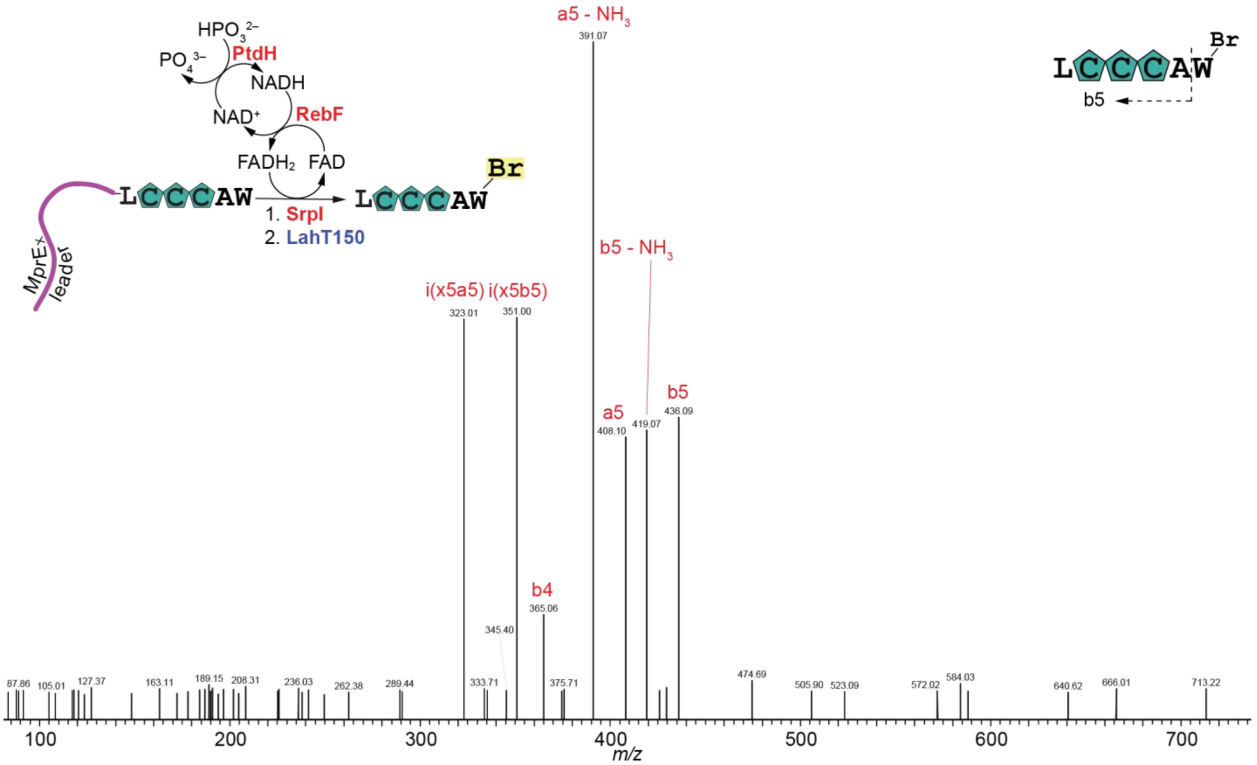

Fig. S8. Structural annotation of the MS^2 fragmentation spectra for the brominated LCCCAW core.

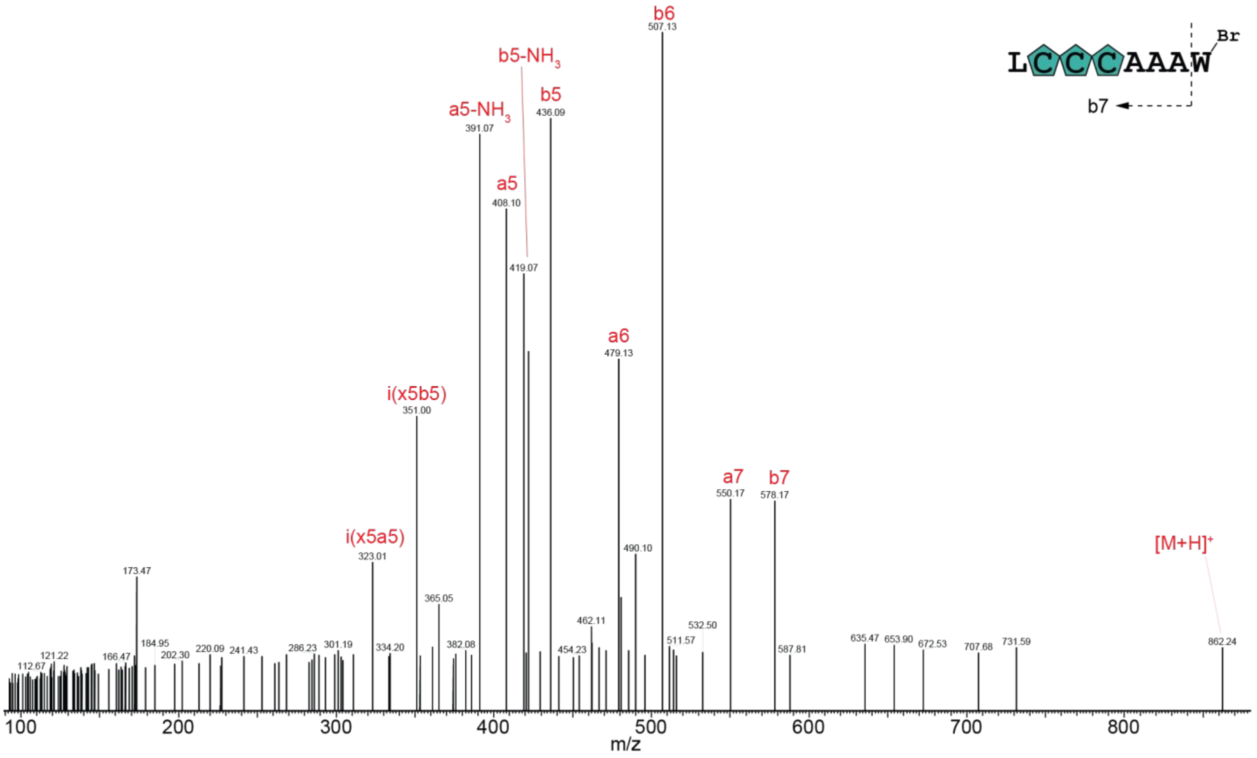

Fig. S9. Structural annotation of the MS^2 fragmentation spectra for the brominated LCCCAAAW core.

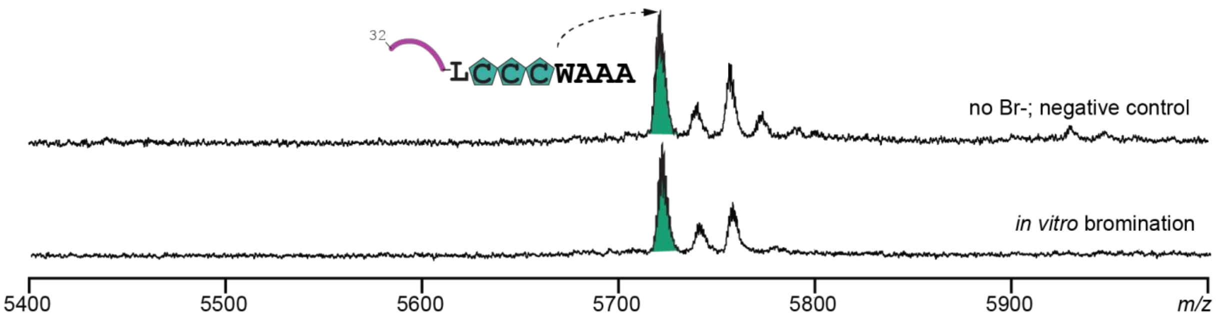

Fig. S10. MALDI-ToF MS spectra for the *in vitro* bromination reaction of MprE$_X$-LCCCWAAA followed by the treatment of the reaction with LysC showing that MprE$_X$-LCCCWAAA is not processed by SrpI.

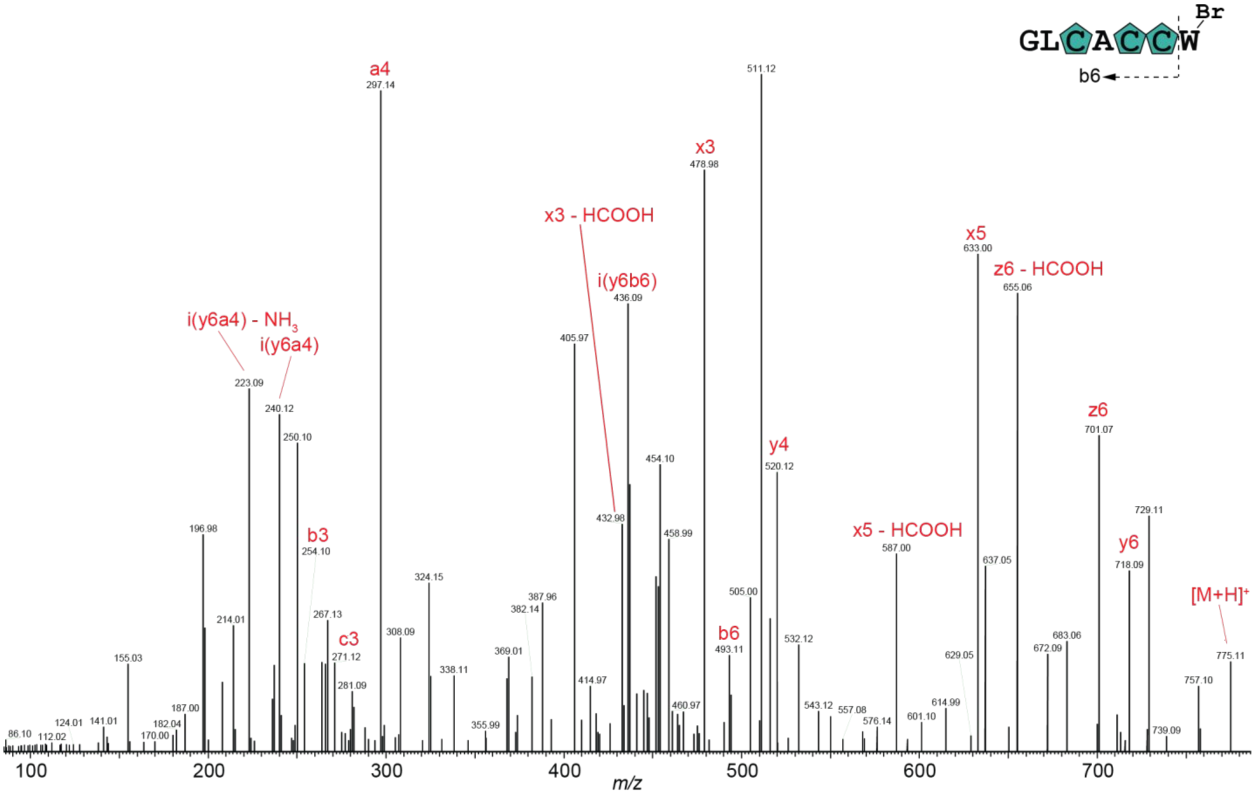

Fig. S11. Structural annotation of the MS² fragmentation spectra for the brominated GL<u>C</u>A<u>CC</u>W core.

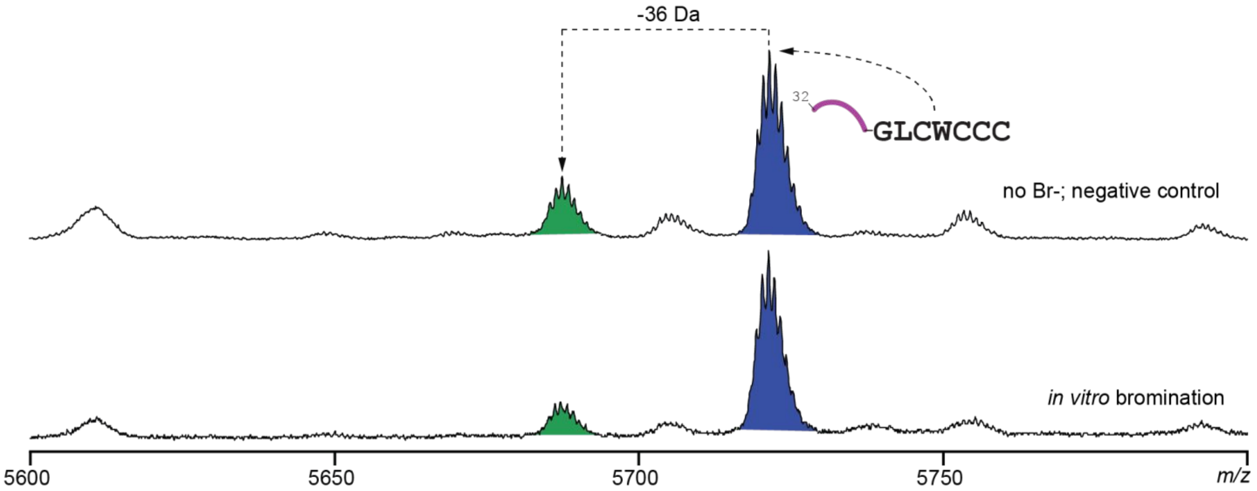

Fig. S12. MALDI-ToF MS spectra for the *in vitro* bromination of MprE$_X$-GLCWCCC followed by the treatment of the reaction with LysC shows that MprE$_X$-GLCWCCC is not processed by SrpI.

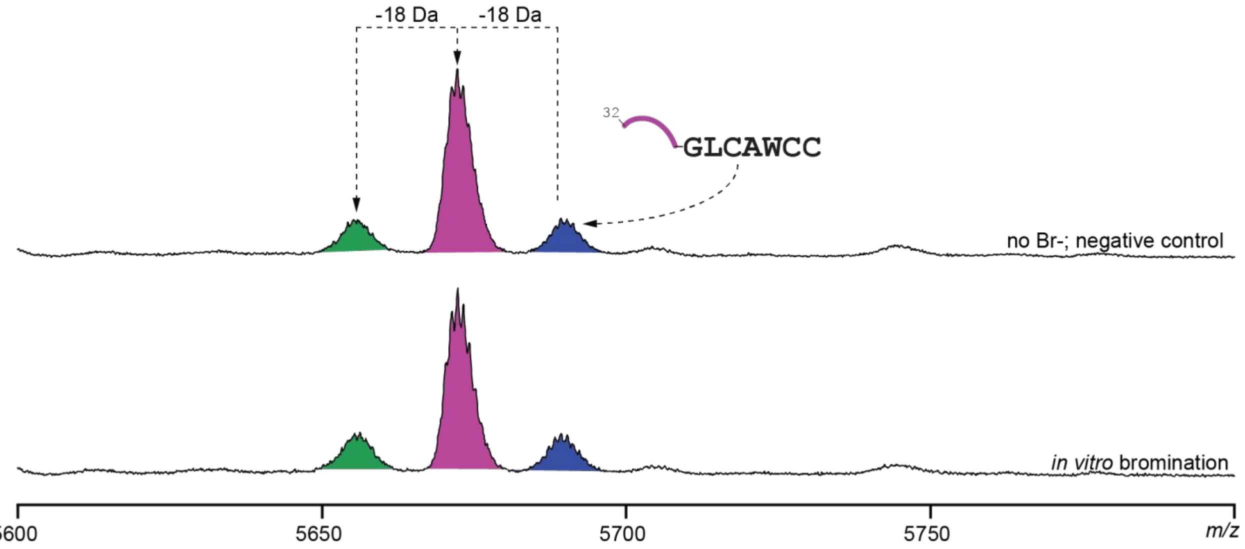

Fig. S13. MALDI-ToF MS spectra for the *in vitro* bromination of MprE$_X$-GLCAWCC followed by the treatment of the reaction with LysC shows that MprE$_X$-GLCAWCC is not processed by SrpI.

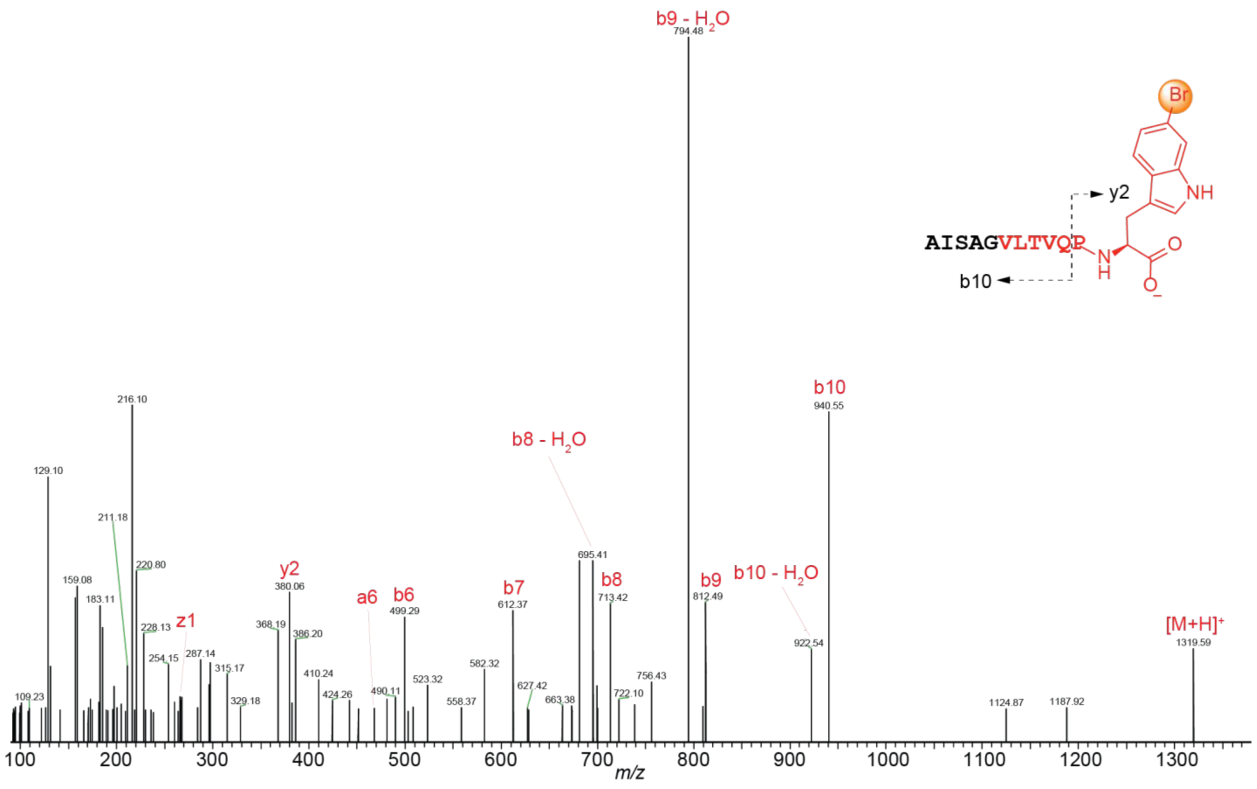

Fig. S14. Structural annotation of the MS^2 fragmentation spectra for the C-terminal fragment containing tumor homing peptide sequence TH2 (VLTVQPW) after *in vitro* halogenation by SrpI followed by the treatment of the product with protease GluC. *m/z* cal.: 1319.60, *m/z* obs.: 1319.59.

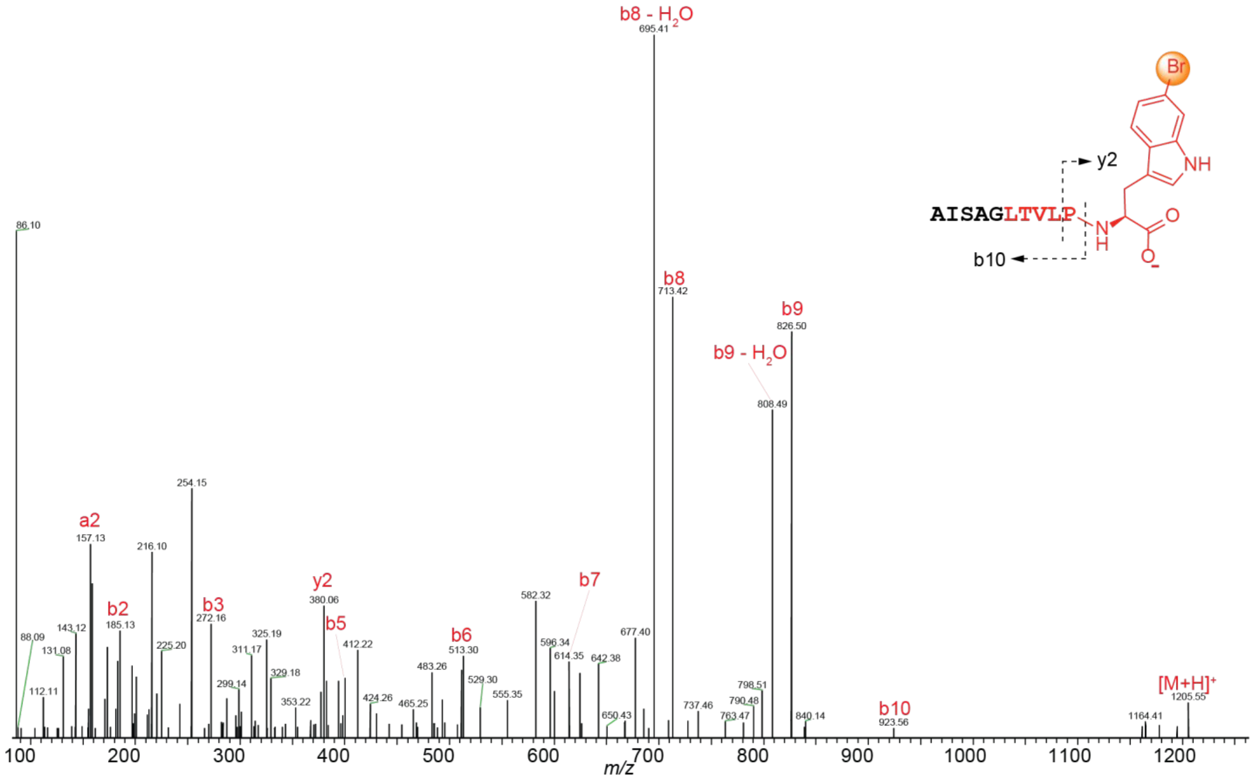

Fig. S15. Structural annotation of the MS[2] fragmentation spectra for the C-terminal fragment containing brominated tumor homing peptide sequence TH1 (LTVLPW) by co-expression of gene encoding SrpE-TH1 with *sprI* followed by the treatment of the purified product with protease GluC. *m/z* cal.: 1205.55, *m/z* obs.: 1205.55

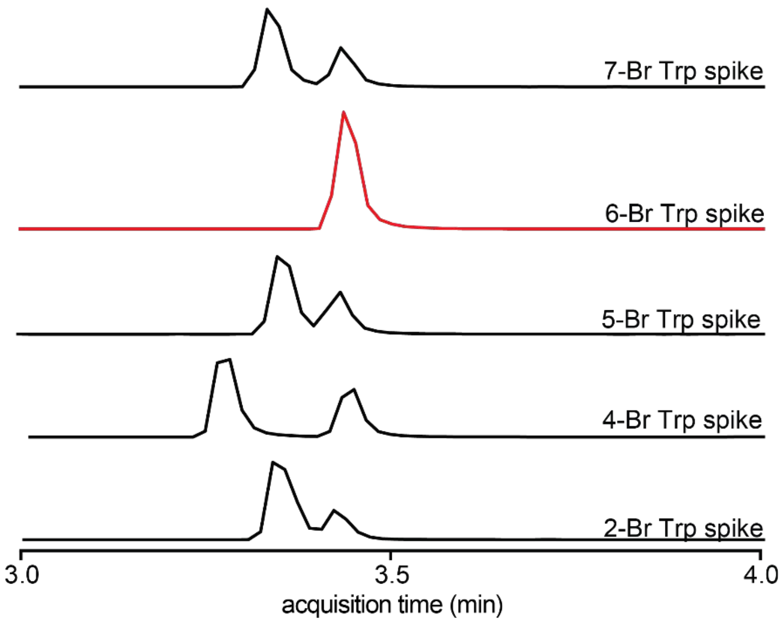

Fig. S16. LC-MS extracted ion chromatograms for *m/z* 283.01 ± 0.1 Da corresponding to bromotryptophan. From bottom to top: co-injection of the carboxypeptidase digestion reaction of brominated SrpE-TH2 with 2-, 4-, 5-, 6-, and 7-bromotryptophan standards. Observation of a single peak upon co-injection with 6-bromotryptophan demonstrates that bromination of the SrpE-TH2 substrate occurred at the indole-6 position.

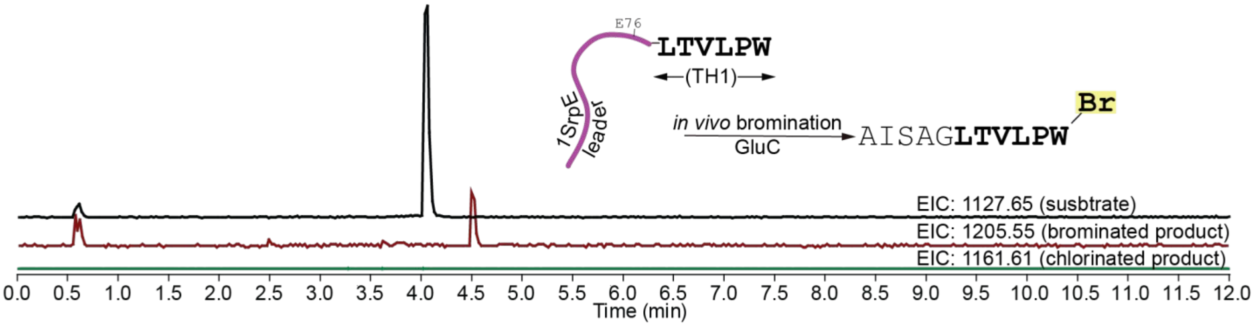

Fig. S17. Scheme for *in vivo* bromination of SrpE-TH1 by SrpI followed by GluC digestion. LC-MS extracted ion chromatograms of the substrate, brominated product, and the conceivable chlorinated product shows that SrpI is strictly specific for incorporation of bromine.

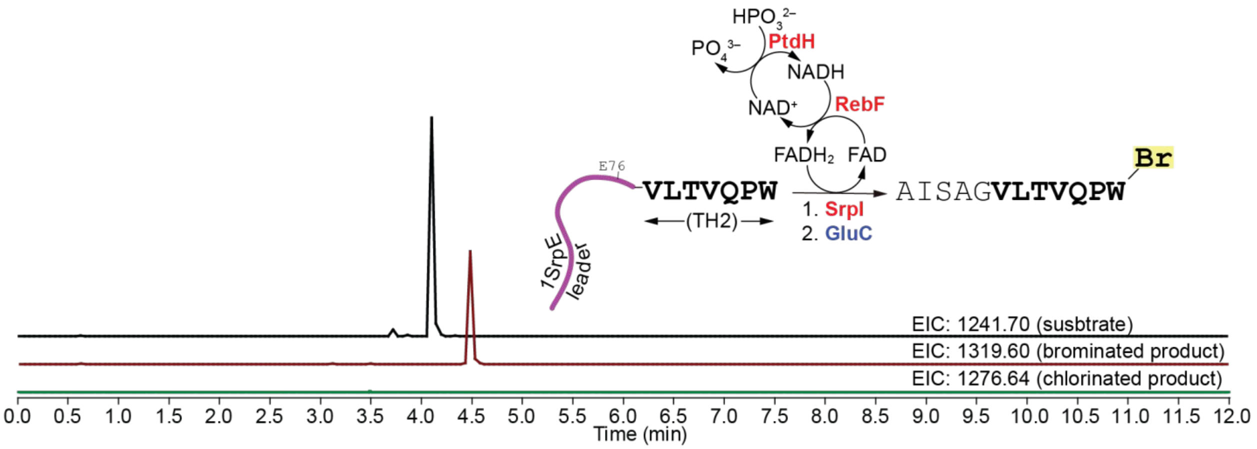

Fig. S18. Reaction scheme for *in vitro* bromination of SrpE-TH2 by SrpI followed by GluC digestion. LC-MS extracted ion chromatograms of the substrate, brominated product, and the conceivable chlorinated product shows that SrpI is strictly specific for incorporation of bromine.

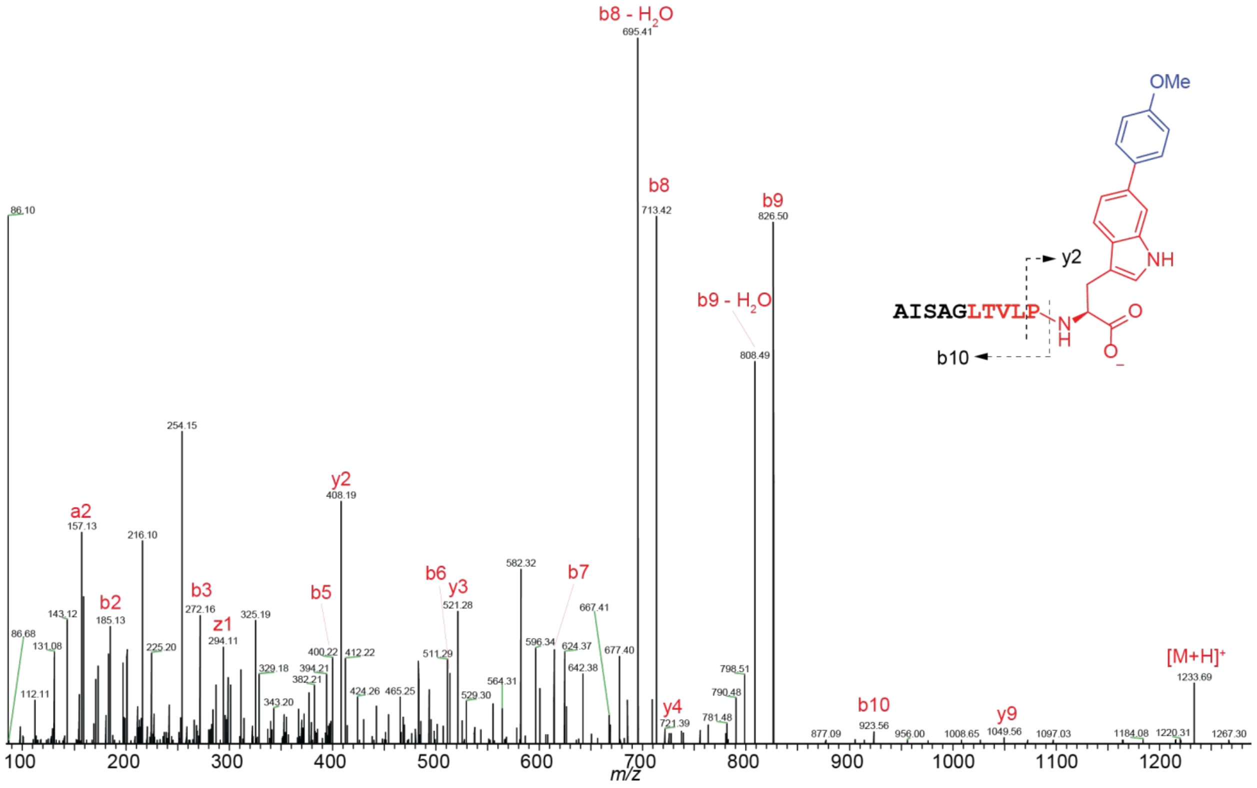

Fig. S19. Structural annotation of the MS^2 fragmentation spectra for TH1-derived Suzuki-Miyaura coupling product, as illustrated. *m/z* cal.: 1233.69, *m/z* obs.: 1233.69.

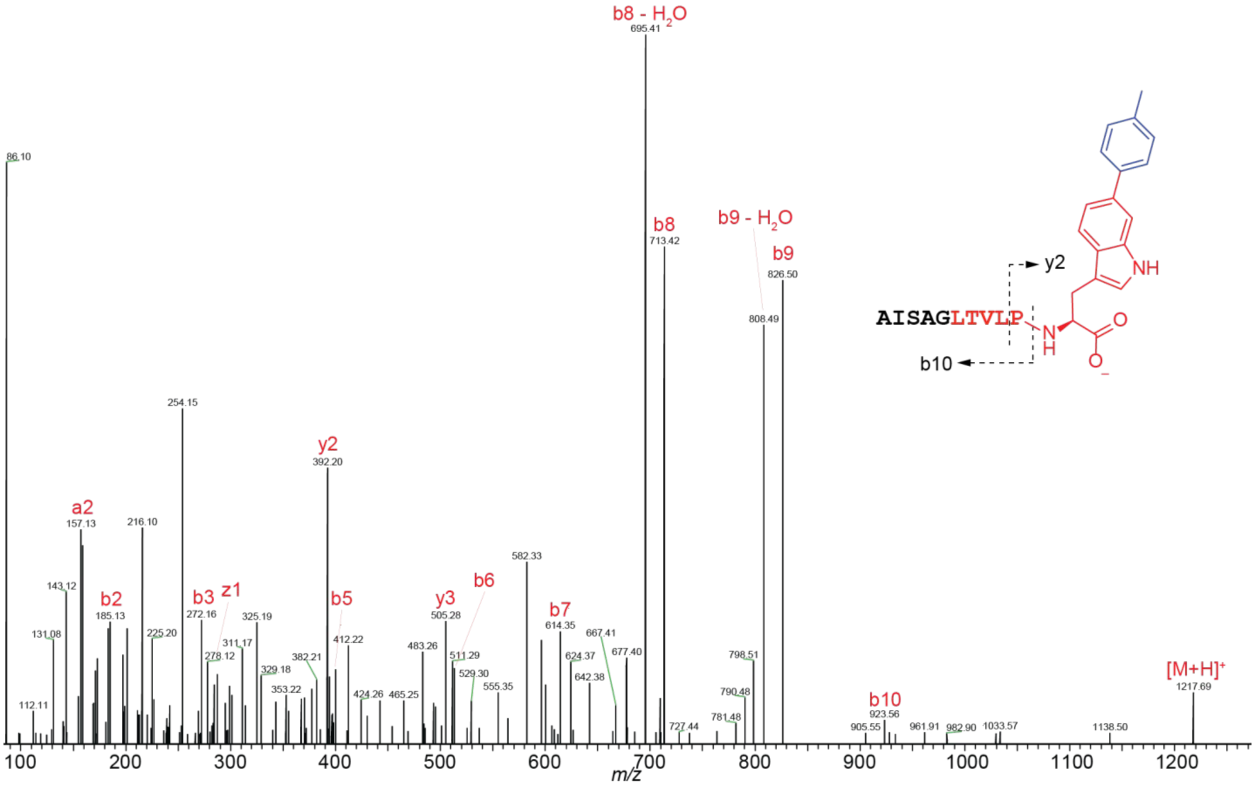

Fig. S20. Structural annotation of the MS^2 fragmentation spectra for TH1-derived Suzuki-Miyaura coupling product, as illustrated. *m/z* cal.: 1217.69, *m/z* obs.: 1217.69.

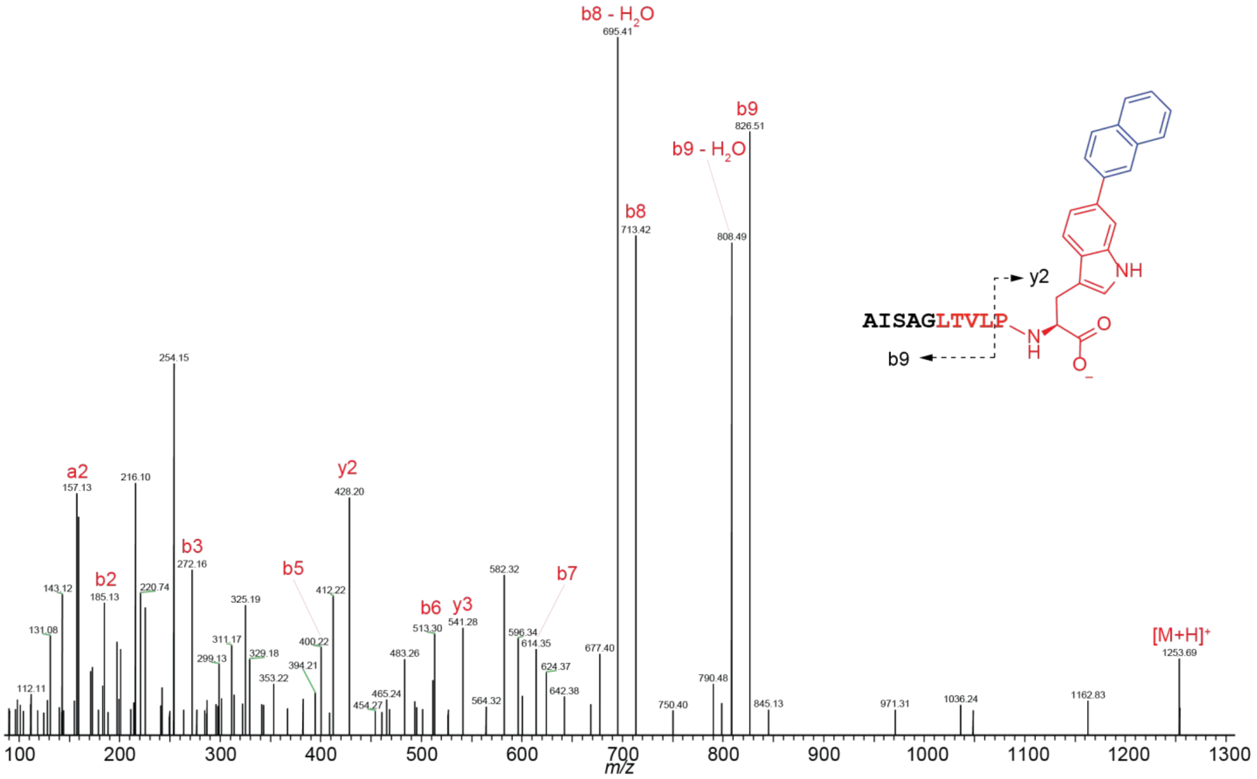

Fig. S21. Structural annotation of the MS^2 fragmentation spectra for TH1-derived Suzuki-Miyaura coupling product, as illustrated. *m/z* cal.: 1253.69, *m/z* obs.: 1253.69.

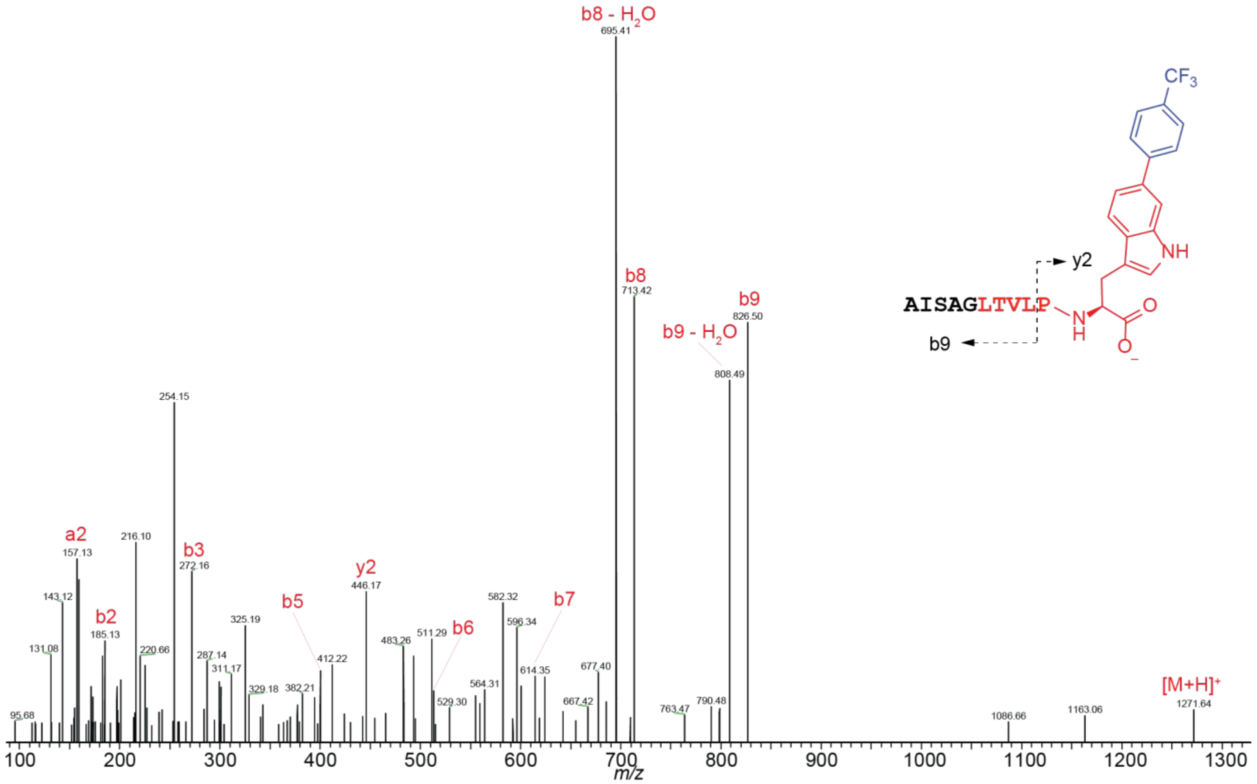

Fig. S22. Structural annotation of the MS² fragmentation spectra for TH1-derived Suzuki-Miyaura coupling product, as illustrated. *m/z* cal.: 1271.66, *m/z* obs.: 1271.64.

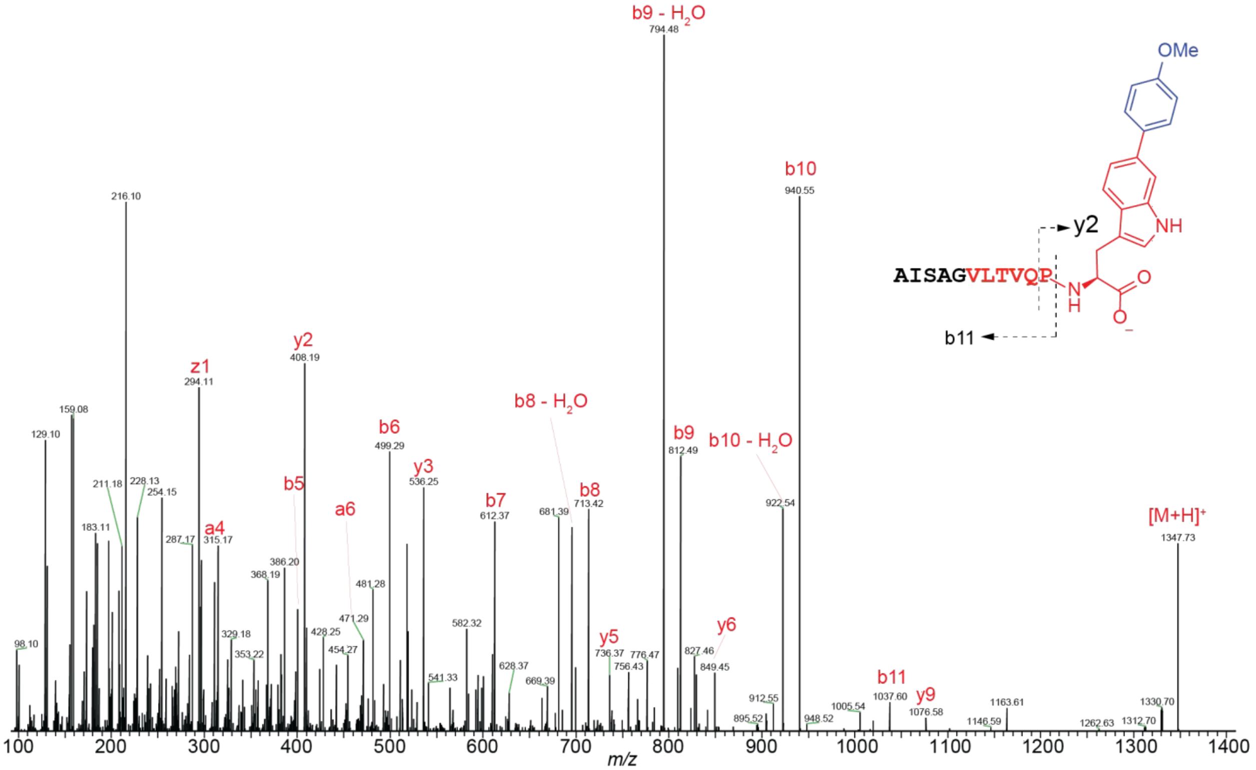

Fig. S23. Structural annotation of the MS² fragmentation spectra for TH2-derived Suzuki-Miyaura coupling product, as illustrated. *m/z* cal.: 1347.73, *m/z* obs.: 1347.73.

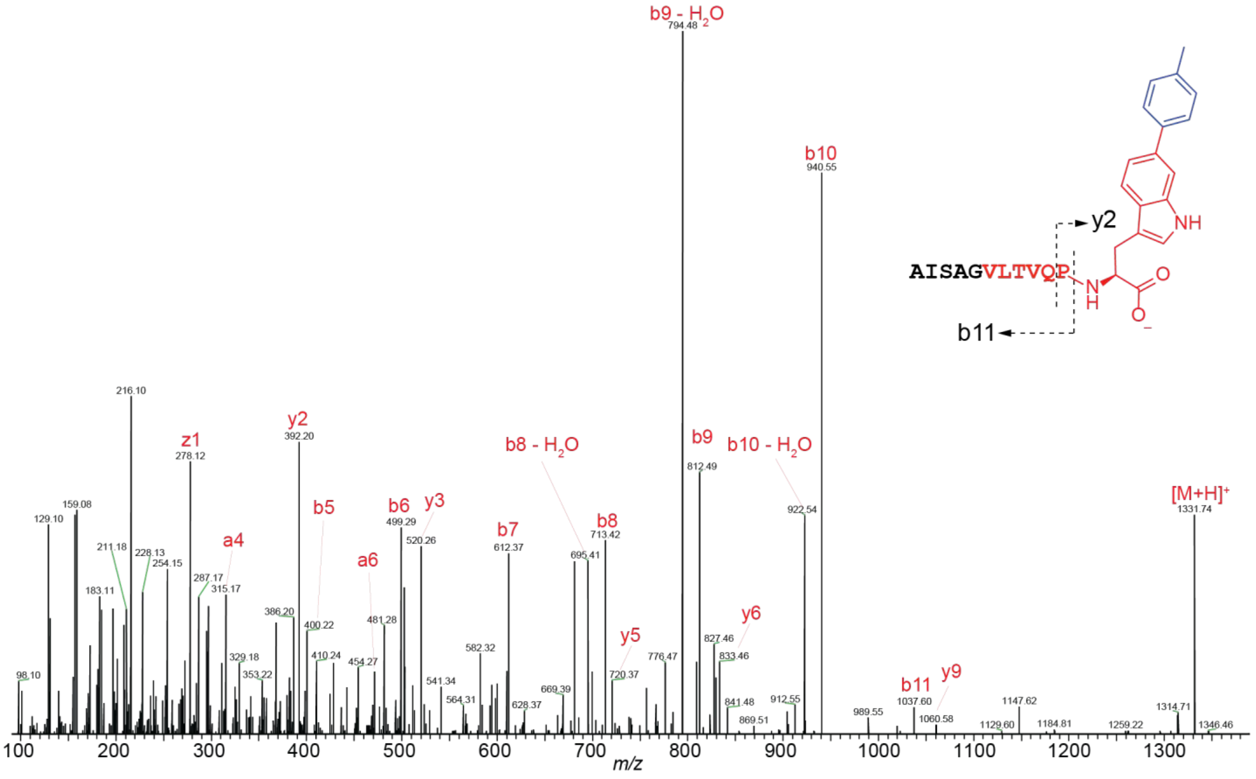

Fig. S24. Structural annotation of the MS^2 fragmentation spectra for TH2-derived Suzuki-Miyaura coupling product, as illustrated. *m/z* cal.: 1331.74, *m/z* obs.: 1331.74.

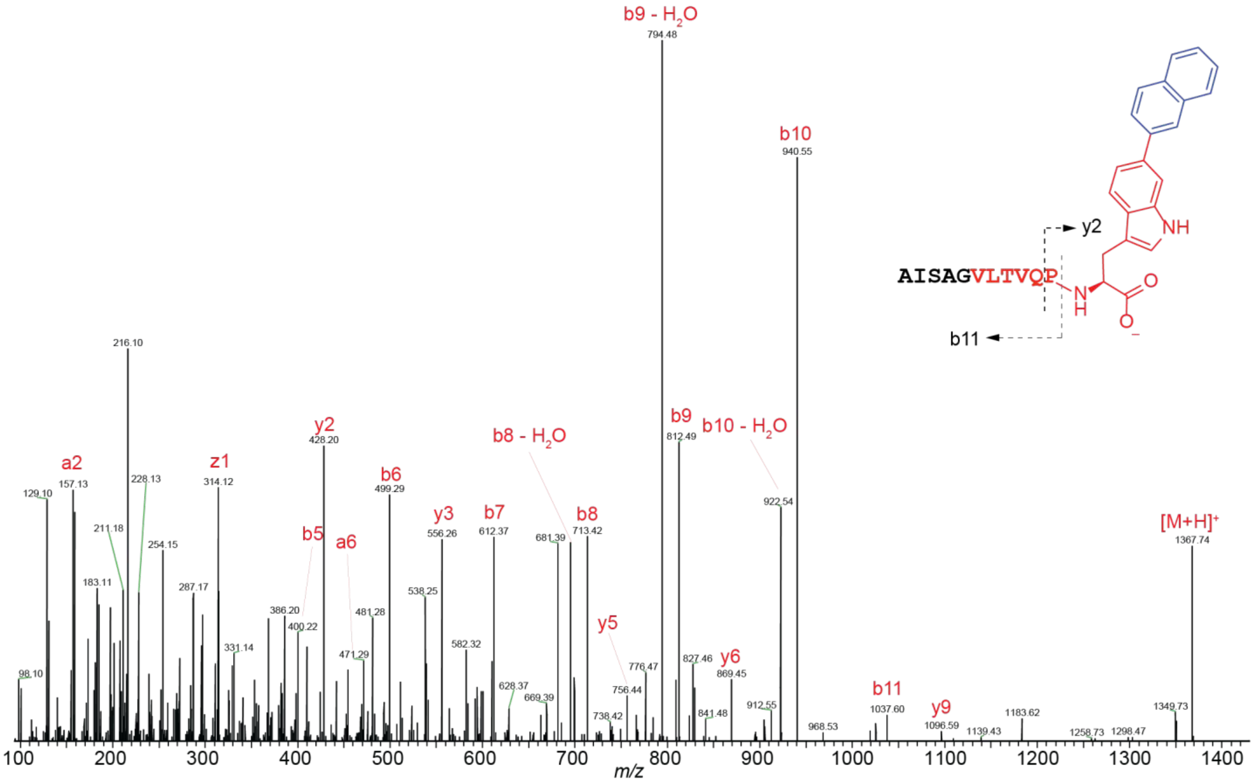

Fig. S25. Structural annotation of the MS^2 fragmentation spectra for TH2-derived Suzuki-Miyaura coupling product, as illustrated. *m/z* cal.: 1367.74, *m/z* obs.: 1367.74.

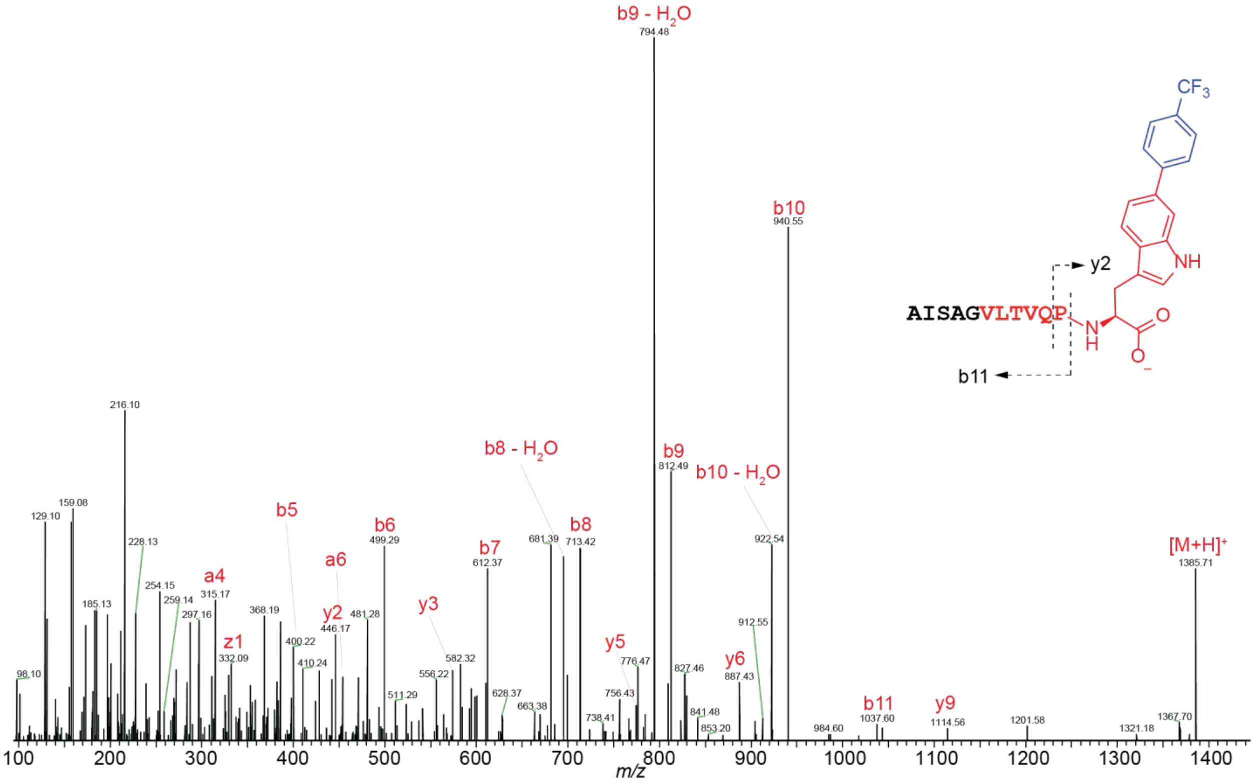

Fig. S26. Structural annotation of the MS^2 fragmentation spectra for TH2-derived Suzuki-Miyaura coupling product, as illustrated. *m/z* cal.: 1385.70, *m/z* obs.: 1385.71.

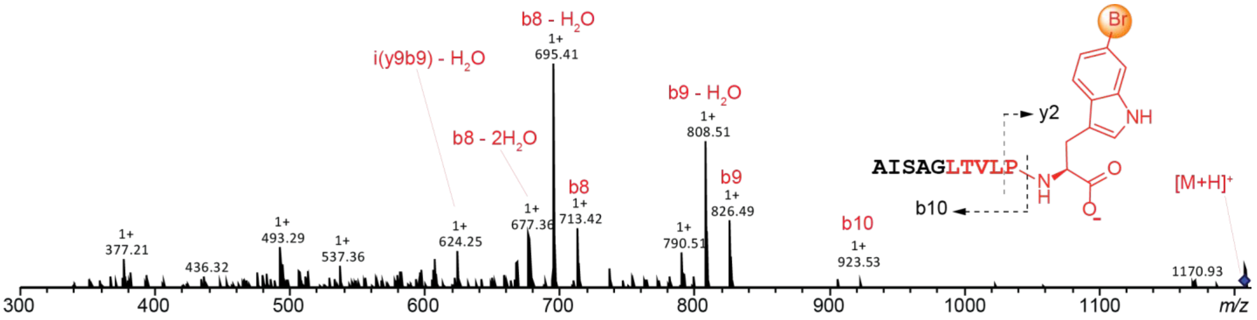

Fig. S27. Structural annotation of the MS[2] fragmentation spectra for the brominated C-terminal fragment of the MBP-SrpE-TH1 substrate delivered by digestion by GluC protease. *m/z* cal.: 1205.55, *m/z* obs.: 1205.55.

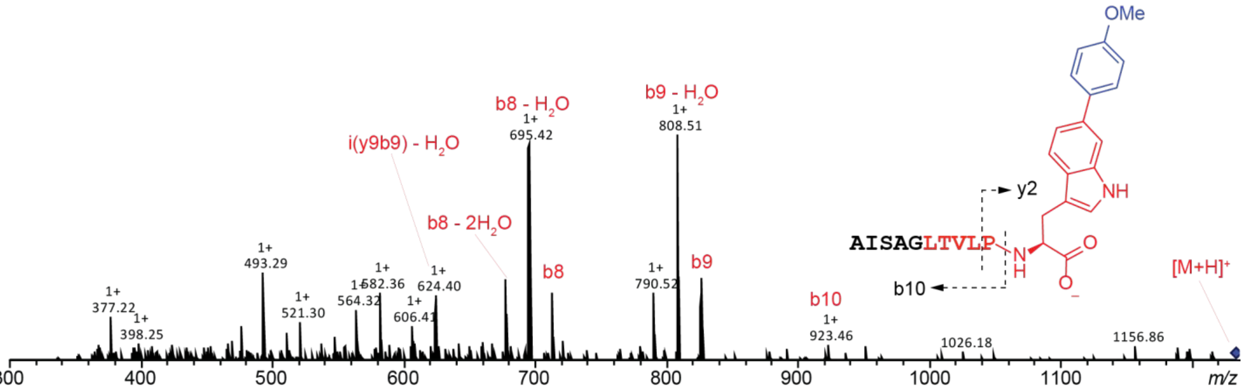

Fig. S28. Structural annotation of the MS[2] fragmentation spectra for the modified C-terminal fragment of the MBP-SrpE-TH1 substrate delivered by digestion by GluC protease. *m/z* cal.: 1233.69, *m/z* obs.: 1233.69.

270

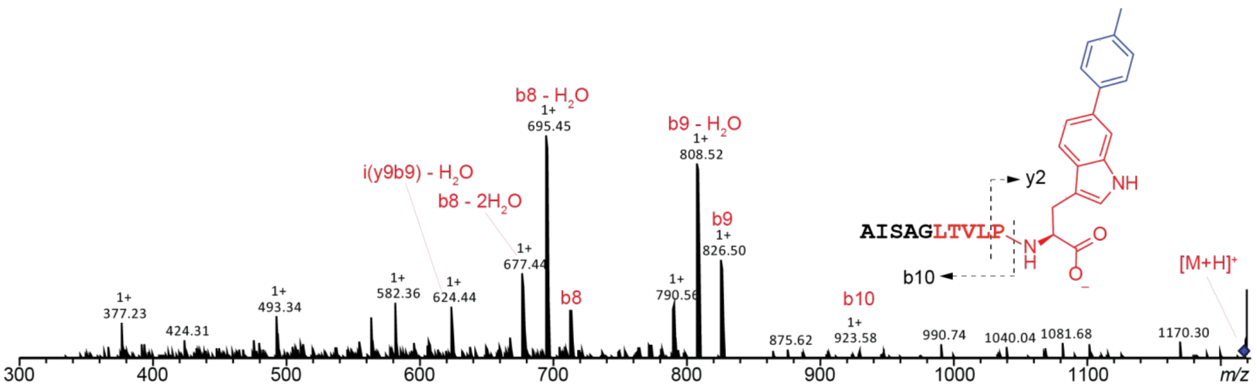

271 **Fig. S29.** Structural annotation of the MS^2 fragmentation spectra for the modified C-terminal fragment of

272 the MBP-SrpE-TH1 substrate delivered by digestion by GluC protease. *m/z* cal.: 1217.69, *m/z* obs.:

273 1217.69.

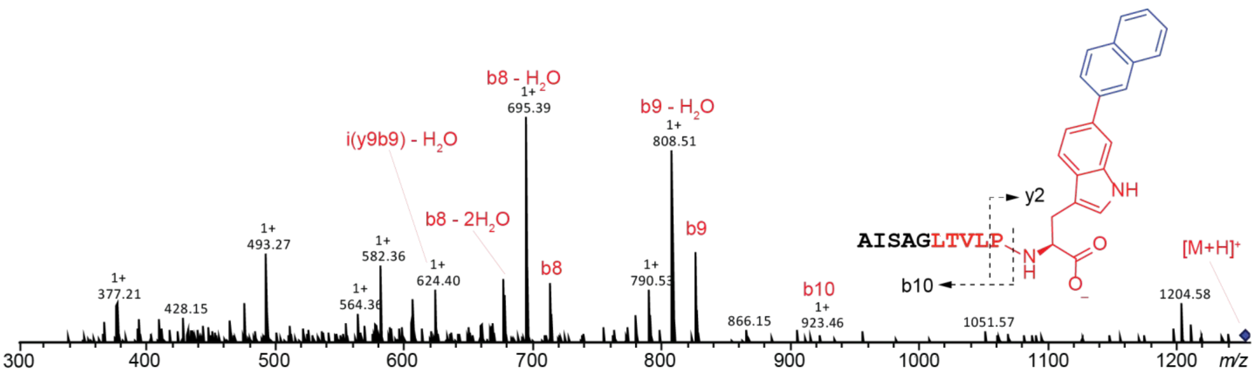

Fig. S30. Structural annotation of the MS[2] fragmentation spectra for the modified C-terminal fragment of the MBP-SrpE-TH1 substrate delivered by digestion by GluC protease. *m/z* cal.: 1253.69, *m/z* obs.: 1253.69.

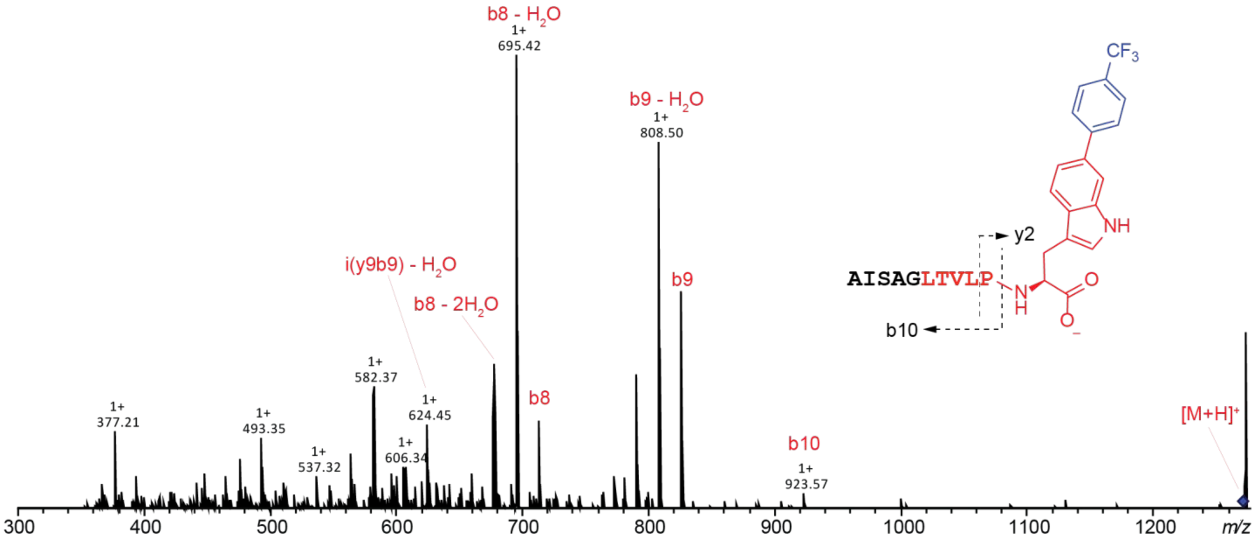

Fig. S31. Structural annotation of the MS^2 fragmentation spectra for the modified C-terminal fragment of the MBP-SrpE-TH1 substrate delivered by digestion by GluC protease. *m/z* cal.: 1271.66, *m/z* obs.: 1271.64.

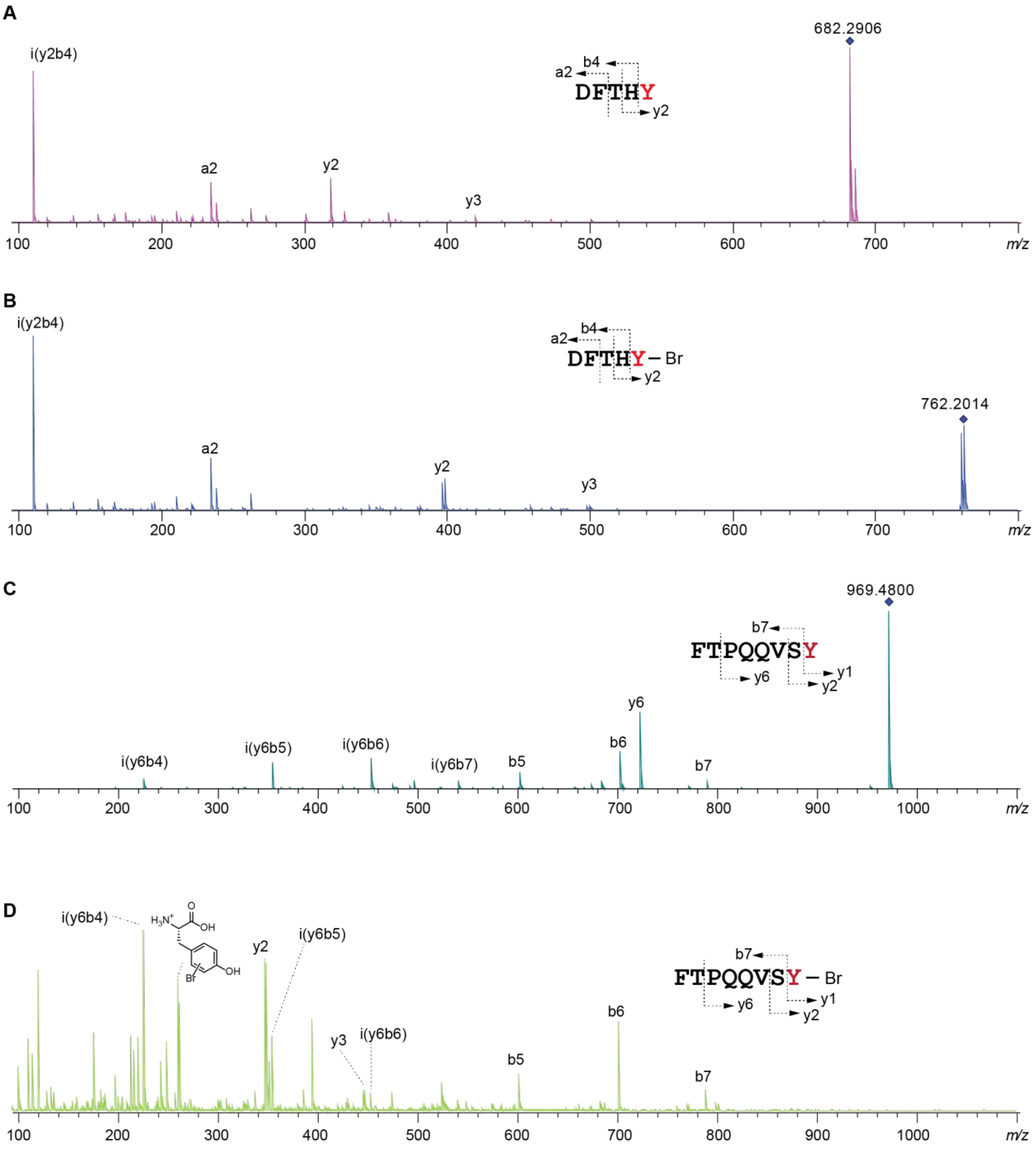

Fig. S32. LC-MS analysis of self-bromination assay products. MS^2 spectrum of the SrpI fragment (**A**) DFTHY and (**B**) the corresponding brominated fragment at *m/z* 682.283 and *m/z* 762.194, respectively. MS^2 spectrum of (**C**) FTPQQVSY and (**D**) the corresponding brominated fragment at *m/z* 969.467 and *m/z* 1047.378, respectively.

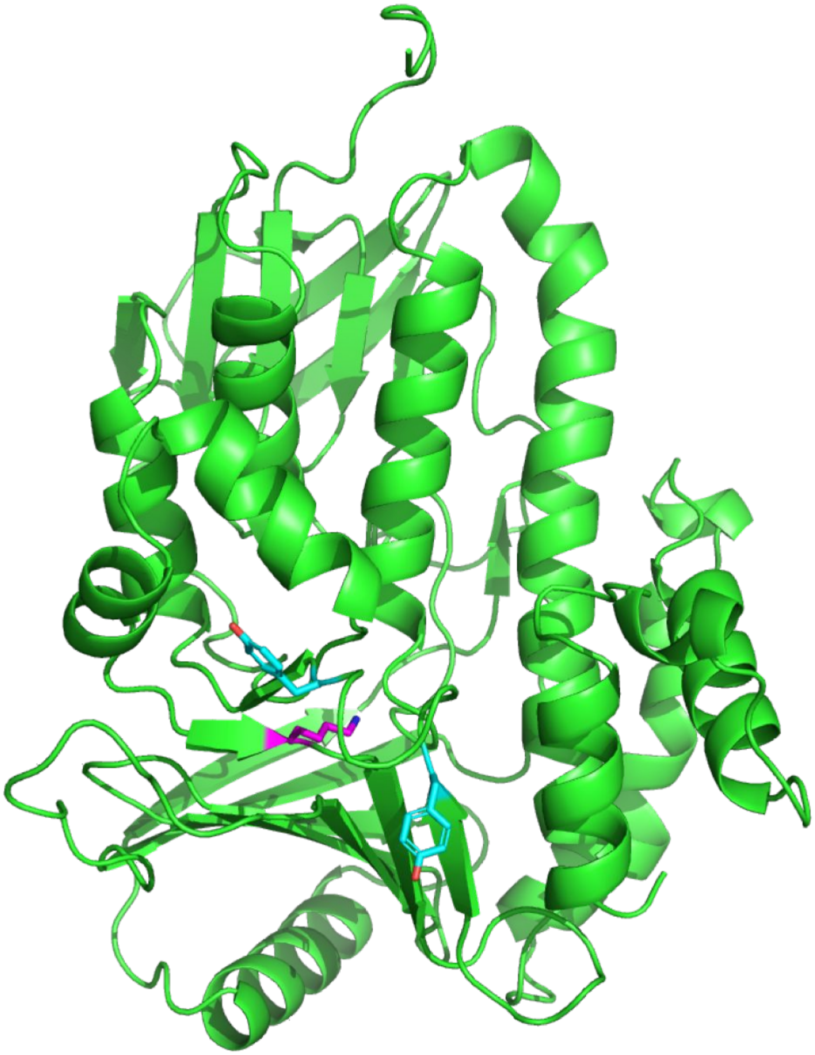

Figure S33. Structural model of SrpI generated by AlphaFold. Catalytic Lys residue is shown in magneta. Two Tyr residues which are self-brominated are shown in cyan.

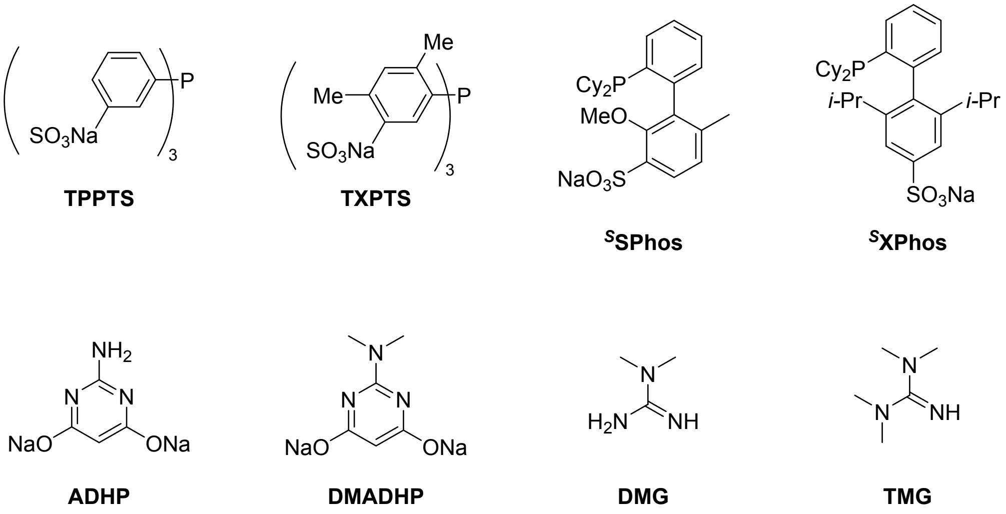

Fig. S34. Selected water-soluble ligands used for aqueous palladium-catalyzed cross coupling reactions.

SUPPLEMENTARY REFERENCES

1. Johannes, T. W.; Woodyer, R. D.; Zhao, H., Efficient regeneration of NADPH using an engineered phosphite dehydrogenase. *Biotechnol. Bioeng.* **2007,** *96* (1), 18-26.

2. Yeh, E.; Garneau, S.; Walsh, C. T., Robust *in vitro* activity of RebF and RebH, a two-component reductase/halogenase, generating 7-chlorotryptophan during rebeccamycin biosynthesis. *Proc. Natl. Acad. Sci. U.S.A.* **2005,** *102* (11), 3960-3965.

3. Nguyen, N. A.; Lin, Z.; Mohanty, I.; Garg, N.; Schmidt, E. W.; Agarwal, V., An Obligate Peptidyl Brominase Underlies the Discovery of Highly Distributed Biosynthetic Gene Clusters in Marine Sponge Microbiomes. *J. Am. Chem. Soc.* **2021,** *143* (27), 10221-10231.

4. Western, E. C.; Daft, J. R.; Johnson, E. M., 2nd; Gannett, P. M.; Shaughnessy, K. H., Efficient one-step Suzuki arylation of unprotected halonucleosides, using water-soluble palladium catalysts. *J. Org. Chem.* **2003,** *68* (17), 6767-6774.

5. Anderson, K. W.; Buchwald, S. L., General catalysts for the Suzuki-Miyaura and Sonogashira coupling reactions of aryl chlorides and for the coupling of challenging substrate combinations in water. *Angew. Chem., Int. Ed. Engl.* **2005,** *44* (38), 6173-6177.

6. Li, J.-H.; Zhang, X.-D.; Xie, Y.-X., Efficient and Copper-Free Sonogashira Cross-Coupling Reaction Catalyzed by Pd(OAc)2/Pyrimidines Catalytic System. *Eur. J. Org. Chem.* **2005,** *2005* (20), 4256-4259.

7. Gao, Z.; Gouverneur, V.; Davis, B. G., Enhanced Aqueous Suzuki–Miyaura Coupling Allows Site-Specific Polypeptide 18F-Labeling. *J. Am. Chem. Soc.* **2013,** *135* (37), 13612-13615.

8. Dumas, A.; Spicer, C. D.; Gao, Z.; Takehana, T.; Lin, Y. A.; Yasukohchi, T.; Davis, B. G., Self-liganded Suzuki-Miyaura coupling for site-selective protein PEGylation. *Angew. Chem., Int. Ed. Engl.* **2013,** *52* (14), 3916-21.

9. Spicer, C. D.; Davis, B. G., Palladium-mediated site-selective Suzuki–Miyaura protein modification at genetically encoded aryl halides. *Chem. Comm.* **2011,** *47* (6), 1698-1700.

10. Li, N.; Lim, R. K. V.; Edwardraja, S.; Lin, Q., Copper-Free Sonogashira Cross-Coupling for Functionalization of Alkyne-Encoded Proteins in Aqueous Medium and in Bacterial Cells. *J. Am. Chem. Soc.* **2011,** *133* (39), 15316-15319.

11. Li, S.; Lin, Y.; Cao, J.; Zhang, S., Guanidine/Pd(OAc)2-Catalyzed Room Temperature Suzuki Cross-Coupling Reaction in Aqueous Media under Aerobic Conditions. *J. Org. Chem.* **2007,** *72* (11), 4067-4072.

12. Latham, J.; Henry, J. M.; Sharif, H. H.; Menon, B. R.; Shepherd, S. A.; Greaney, M. F.; Micklefield, J., Integrated catalysis opens new arylation pathways via regiodivergent enzymatic C-H activation. *Nat. Commun.* **2016,** *7*, 11873.
